# A computational framework to study EGFR signaling distribution in egg chambers during dynamic interactions between soma and germline

**DOI:** 10.1371/journal.pcbi.1013802

**Published:** 2025-12-29

**Authors:** Nastassia Pouradier Duteil, Nicole T. Revaitis, Mathew G. Niepielko, Eric A. Klein, Nir Yakoby, Benedetto Piccoli

**Affiliations:** 1 Center for Computational and Integrative Biology, Rutgers, The State University of NJ, Camden, New Jersey, United States of America; 2 Department of Biology, Rutgers, The State University of NJ, Camden, New Jersey, United States of America; 3 Department of Mathematical Sciences, Rutgers, The State University of NJ, Camden, New Jersey, United States of America; University of Southern California, UNITED STATES OF AMERICA

## Abstract

Forming organs requires the appropriate distribution of spatiotemporal signals leading to tissue patterning and morphogenesis. Advances in genetic tools contributed to our understanding of cell signaling and their associated genes. Yet, due to technical challenges, the contribution of dynamic morphological transformations of tissues during organ formation remains widely unexplored. Here, we develop a new mathematical approach to understand the variables that shape the dynamic distribution of ligand and signaling. We use the TGF-α-like ligand Gurken (GRK) and the activation of the epidermal growth factor receptor (EGFR) during *Drosophila* oogenesis to build the model. Our model accounts for GRK secretion from a moving source, its diffusion in the perivitelline space, and the activation of EGFR in the overlaying follicle cells. Furthermore, we also capture the rapid growth of the oocyte, which was a major challenge to integrate into a model. We modeled the dynamic distribution of GRK and EGFR activation by a series of mathematical equations. We used this model to study how perturbations of the egg chamber’s morphological evolution impact cell signaling, which could not be achieved via genetic perturbation. We found that the relative movement of the follicle cells and the oocyte contributes to the distribution of EGFR signaling activation.

## Author summary

Cell differentiation is a key process in an organism’s development, and it relies on spatiotemporal information provided by the anisotropic and time-dependent distribution of signals. For this reason, understanding the pattern formation of signal within a growing organism is central to understanding cell differentiation, which in turn leads to organ formation. *Drosophila* oogenesis serves as a model framework to study the coordination of many processes, including cell migration, diffusion of signal, and reaction pathways within the cells. However, isolating each of these mechanisms to understand its role in this complex interaction can be difficult, or even unfeasible, from an experimental point of view, because of the lethality of some perturbations. To overcome this limitation, we propose a comprehensive mathematical model which includes the interacting pieces, and which can serve as a complementary tool to understand the role of each mechanism in the global pattern formation via numerical simulations.

## Introduction

Organ formation requires the coordination between cell proliferation and differentiation. During this process, cells depend on spatiotemporal information to identify their location in the tissue and designated functions [1,2]. A small number of cell signaling pathways control this process; cells respond by adjusting their position depending on concentration gradients of ligands [3,4]. The extracellular signal is transmitted to the cell nucleus by intracellular components, which regulates gene expression that patterns the tissue. Consequently, the initially uniform tissue transforms to a non-uniform field of cells that can form morphologies [5,6]. Advances in genetic perturbations mediated the discovery of interacting networks and the roles of morphological changes during tissue development [7,8]; yet, some perturbations terminate developmental processes, including halting cells’ movement and compartments’ growth, thus restricting our ability to study the contributions of changes in cellular compartments to signaling.

Mathematical models can provide solutions to overcome experimental challenges. Models for tissue patterning initially demonstrated that spatial heterogeneities can arise from the reaction and diffusion of competing chemical substances, also known as morphogens [9]. There exists a wide variety of models for the deformation or growth of evolving surfaces [10–13]. Modeling was used to explain periodic patterning in various organisms, such as in the marine angelfish [14], and digit formation during limb development [15]. Interestingly, it was recently suggested that domain growth and tissue curvature have a non-negligible effect on the diffusion of morphogen/ligand, cell signaling, and tissue patterning [14–21]. In addition, several studies explored the growth driven by morphogen concentration, as well as the coupling between growth and morphogen diffusion [22–26]. At the same time, there exist many models to describe collective cell migration, driven or not by a chemoattractant [27,28]. However, to the best of our knowledge, models that couple the dynamics of morphogen distribution over a growing manifold with movement of cells during organogenesis are yet to be available, which is the mathematical challenge we address here.

**Fig 1:**
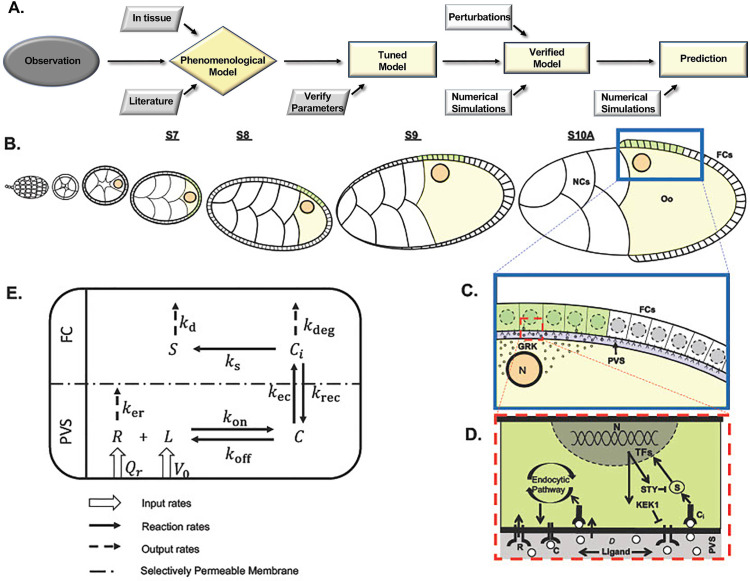
A. Workflow for the construction, tuning, verification and use of the model. **B-E:** Schematic of the mechanisms used for defining the parameters of the model. **B:** The oocyte nucleus has a dynamic localization relative to the follicle cells during Stages 7 to 10A of oogenesis (top). GRK is localized around the oocyte nucleus, diffuses in the perivitelline space and binds to the EGFR located on the surface of the overlying follicle cells (**C**, blue inset). Internalization of GRK sets off the RAS/RAF/MEK signaling cascade as well as the production of inhibitors that act as negative feedback (**D**, red inset). **E**: Reaction pathway between ligand and other molecules after binding to EGFR. Names are explained in Table 1. The parameters considered in our model consist of three movements during stages 7-10A of oogenesis. These movements include: 1) growth, 2) the transient source of GRK in the developing oocyte, 3) the rearrangement of the FCs from cuboidal to columnar and stretched.

The *Drosophila melanogaster* oogenesis is a well-described process, where defined cell signaling pathways pattern the moving follicular epithelium over a growing oocyte; these cells will later form the eggshell [29–31]. Specifically, the egg chamber, the precursor of the mature egg, is comprised of 16 germ cells engulfed by a monolayer of follicular epithelium. Over the 14 morphologically distinct stages of oogenesis [32], one germ cell becomes the oocyte and the other 15 become the nurse cells, providing for the growing oocyte. At Stage 7 (S7), the oocyte is small, and its nucleus is positioned at the posterior end (Fig 1B). At stage 8 (S8), the oocyte nucleus is anchored to the dorsal anterior of the growing oocyte. Concomitantly, the follicle cells (FCs) compact towards the posterior, engulfing the oocyte as columnar epithelium by the end of S9 (Fig 1B) [31,33,34].

**Table 1:** Parameters of the model.

Symbol	Parameter	Value or Range	Source
***L***_***FC***_	Length of columnar FCs	Variable (See Fig. S1)	This study
***L***_***AP***_	Length of egg chamber	Variable (See Fig. S1)	This study
***L***_***Oo***_	Length of oocyte	Variable (See Fig. S1)	This study
***L***_***DV***_	Width of oocyte	Variable (See Fig. S1)	This study
***H***	Perivitelline space thickness	0.5 µm	This study
***D***	Rate of Diffusion	36-360,000 µm^2^ hr^-1^	Pribyl et al., 2003
***k***_***ec***_	Rate of internalization of complex	6 hr^-1^	Pribyl et al., 2003
***k***_***on***_	Rate of Ligand-Receptor binding	6 x 10^22^ – 6 x 10^25^ mol^-1^ µm^3^ hr^-1^	Pribyl et al., 2003
***k***_***off***_	Rate of Ligand-Receptor dissociation	6 hr^-1^	Pribyl et al., 2003
**R**_**0**_	Free receptor per surface area in the absence of ligand	6.7 x 10^-22^ mol µm^-2^	Pribyl et al., 2003
***k***_***er***_	Receptor internalization independent of ligand	0.6-6 hr^-1^	Pribyl et al., 2003
***α***_***rec***_	Fraction of recycled receptor	0.45-0.7	Sigismund et al., 2008
***α***_***deg***_	Fraction of degraded receptor	0.3-0.55	Sigismund et al., 2008
k_rec_	Receptor recycling rate	2.3 hr^-1^	Sigismund et al., 2008
k_deg_	Receptor degradation rate	2.3 hr^-1^	Sigismund et al., 2008
k_d_	dpERK degradation rate	2.3 hr^-1^	Pinilla-Macua et al., 2016
*V*_*0*_	Initial flux of ligand from the oocyte	2x10^-21^ mol µm^-2^ hr^-1^	Pribyl et al., 2003
*Q*_*r*_	Receptor production rate	4x10^-22^ mol µm^-2^ hr^-1^	Pribyl et al., 2003
*k*_*s*_	dpERK production rate	1 hr^-1^	Arbitrary

Drosophila oogenesis is governed by numerous cell signaling pathways [35–42], including the epidermal growth factor receptor (EGFR) [38,43,44]. The TGF-alpha-like ligand *gurken* (*grk*) is transcribed by the nurse cells, transferred to the oocyte, and positioned around the oocyte nucleus. Thereafter, it is translated and secreted to the perivitelline space, and signals through an expressed EGFR in the overlaying FCs [37,38,44]. The appropriate levels of EGFR activation during oogenesis are essential for egg development, and the absence of GRK generates eggs that lack anterior-posterior (AP) and dorsal-ventral (DV) axes [35,36,41,45–47]. Egg chamber development provides dynamic position of ligand source (around the oocyte nucleus), and growing oocyte while the FCs (the ligand destination) move towards the posterior end. Hence, our system is excellent for developing a complex model that examines the impact of compartments’ interactions on EGFR signaling.

The Shvartsman Lab formed an elegant model that accounts for EGFR signaling in the FCs [48,49], however, the model is at steady-state and does not account for the dynamics of EGFR activation and the growing egg chamber. To address these issues, we developed a novel mathematical framework. Our model uniquely combines a time-varying family of Riemannian manifolds, a reaction-diffusion system on the manifolds via the Laplace-Beltrami operators [50], and advection terms for the relative movements of different domains. For numerical implementation, we used finite difference numerical schemes for partial and ordinary differential equations, and domain decomposition and cubed-sphere discretization to deal with the growing manifold shape.

Our model takes into consideration the dynamic position of GRK in the growing oocyte, its secretion and diffusion in the perivitelline space, and the relative movement of the FCs over the oocyte [38,39,44,51]. The role of the diffusion and reaction of molecules in pattern formation has been studied in many contexts, including Drosophila oogenesis [48,49]. Moreover, the effect of domain growth on pattern formation has recently been the focus of several theoretical works [18–21]. However, to our knowledge, there exist relatively few examples of mathematical models designed for a specific biological system aiming to study the effect of its morphological evolution on signal distribution, coupling numerical simulations and experimental data.

Another important component of our model concerns the relative movement of the FCs and of the oocyte, and its effect on pattern formation. As stressed in [52], the role of cell movement in pattern formation is mostly unappreciated in the literature, although there are several examples of biological systems in which it seems to play an important role (see [52] and references within). We chose to include the movement of the FCs in our model and explore the role of this shift in the final signal distribution.

The tuned model was then used as a tool to examine the effects of perturbations of the egg chamber compartment’s morphological evolutions, which we failed to perform experimentally due to lethality. Our model suggests a new mechanism shaping EGFR activation in the FCs that depends on the relative movement of FCs over the oocyte. Our approach is an innovative mathematical framework that is amenable to study other growing organs with known source(s) of the ligand(s).

## Results

To clarify our approach, we generated a workflow for the construction, tuning, verification and use of the model (Fig 1A). From quantitative imaging, we first built a *phenomenological* model, by incorporating key elements and formulating them in the framework. The numerous model parameters were then tuned using both direct and indirect experimental measurements, and data from the literature. We then proceeded to verify the model by comparing its performance to the wild-type and genetic perturbed settings with experimental results. Lastly, we used the model to predict the effect of morphological perturbations, including stopping the posterior movement of the follicular epithelium, which failed experimentally due to lethality.

### Construction of the mathematical framework

We aim to build a mathematical model capable of integrating the interactions among heterogeneous developmental mechanisms. Most notably, the model takes into consideration two categories of phenomena: mechanistic changes at the tissue level (evolving geometry) and reactions with other entities at the molecular level (ligand concentration, receptor activation, negative feedback). The resulting equation describing the evolution of the ligand and signal densities is a transport-reaction-diffusion system, with additional terms coming from the mechanistic changes.

Our framework considers the interplay of various mechanisms in a complex space divided into several different physical compartments:

- the diffusion of molecules in a (or several) compartment(s).
- the movement of the source of molecules.
- the growth of the compartments.
- the relative movement of compartments.
- the reaction between molecules.

To anchor this general framework in a biological setting, we focus on the dynamic distribution of GRK during *D. melanogaster* oogenesis, a well-documented model system to study cell signaling, tissue patterning, and morphogenesis [29,31,35,53,54]. We take into consideration the secreted ligand GRK from around the oocyte nucleus into the perivitelline space, a narrow region enclosed between the oocyte and the overlaying FCs (Fig 1B and 1C). The position of the oocyte nucleus is dynamic. Up to stage 7 (S7), it is anchored to the posterior end. At S8, the nucleus is anchored to the cortex of the oocyte and remains there throughout egg chamber growth. Since GRK is no longer localized after S10A, we focus on the time between S7 to S10A (Fig 1B). During these states, the FCs become columnar towards the posterior, generating an opposing movement relative to the secreted GRK. In the anterior, about 55 cells engulf the nurse cells, by generating squamous cells called stretched cells [32,33,55–57]. At the same time, the egg-chambers’ dimensions increase four-fold, and the oocyte grows from occupying only a small region at the posterior of the egg chamber (S7) to occupying half of the egg-chamber (S10A).

Taken together, we identified three mechanistic changes that may affect the distribution of the ligand: 1) the dynamic position of the oocyte nucleus; 2) the growth of the oocyte and egg-chamber; and 3) the relative movement of the FCs over the oocyte nucleus. Additionally, we account for the diffusion of GRK (Fig 1D) and interaction with EGFR in the overlaying FCs. Furthermore, we account for the negative feedback by Kekkon (KEK) and Sprouty (STY). The overall interactions in the model are summarized in Fig 1E.

We explain the model below step by step. Due to the complexity of the model, we build it incrementally, gradually adding in the mechanisms that contribute to the full picture. The resulting full system of equations, describing the diffusion of ligand in a growing perivitelline space, and its interactions with receptors to produce signal, is presented in Equation (5).

1 – ***Diffusion of ligand in the perivitelline space***

The main assumption of our model is that the width of the perivitelline space is negligible compared to the other dimensions of the system (such as the anterior-posterior and dorsal-ventral dimensions of the egg-chamber). This assumption agrees with previous publications [48,58]. Hence, we consider that GRK diffuses on a curved two-dimensional manifold, which approximates the perivitelline space (S3 Fig in S1 Text). Following Goentoro et al., 2006, we model this surface by a prolate spheroid, that we parameterize by a two-dimensional space variable x=(η, θ) (S3 Fig in S1 Text).

Then, if we omit in this first step the growth of the perivitelline space, the diffusion of the ligand, L, is given by the following partial differential equation:


$$\frac{\partial L(t,x)}{\partial t}=D\;\Delta_{\text{surf}}L(t,x)$$ (1)

where $\Delta_{\text{surf}}$ represents the Laplace-Beltrami operator of the surface (which for now is supposed to be fixed) and D is the diffusion rate. More information on diffusion over manifolds and the Laplace-Beltrami operator is found in [50].

2 – ***Movement of the oocyte nucleus***

The position of the oocyte nucleus, which acts as a source of ligand, changes from being a posterior end position to the dorsal anterior of the oocyte. We take this movement into account in the source function V(t,x) which represents the time and space-dependent flux of ligand. Adding this source, the diffusion equation with source, still on a constant surface, rewrites as:


$$\frac{\partial L(t,x)}{\partial t}=D\;\Delta_{\text{surf}}L(t,x)+V(t,x),$$ (2)

where the function V will be given by experimental measurements (S2 Fig in S1 Text).

3 – ***Growth of the egg chamber***

Between S7 to S10A, the dimensions of the egg chamber increase by a factor of 4 along the AP axis and by a factor of 3 along the DV axis (S1 Fig in S1 Text). The growing size of the egg chamber/oocyte may impact signaling distribution over developmental time. Taking growth into account, we added a transport term to the equations. This transport is given by the flow of a vector field  v (calculated from measurements of AP and DV (S1 Fig in S1 Text) that transforms the dimensions of the prolate spheroid, which represents the oocyte (S3 Fig in S1 Text). During growth, the diffusion operator becomes time-dependent due to the changing surface geometry over time. To highlight this time dependence, we change notations and, from here onwards, we denote the Laplace-Beltrami operator of the surface at time t by $\Delta_{t}$ (replacing the previously fixed operator $\Delta_{\text{surf}}$). We refer the reader to [59], in which we introduced the time-varying Laplace-Beltrami operator. Considering the growth of the egg-chamber, the concentration of ligand now satisfies the following PDE:


$$\frac{\partial L(t,x)}{\partial t}+\nabla \cdot \left(v(t,x)\;L(t,x)\right)=D\;\Delta_{\text{t}}L(t,x)+V(t,x),\;$$ (3)

where ∇· denotes the divergence operator.

4 – ***Reactions between ligand and other molecules***

After secretion, GRK molecules diffuse in the perivitelline space (Fig 1C) and bind to the EGF receptors in the overlying FCs [51]. Many parameters at the interface of the perivitelline space and the FCs are considered to impact the distribution of GRK. In general, we will denote by $P=(P_{1},...,\;P_{m})$ a family of molecules (that will be specified in the next steps), other than EGFR, that interact with the ligand through some reaction terms denoted$\;{ℛ}_{1}$ and ${ℛ}_{2}$ (that will also be specified later). Assuming that the other molecules are also affected by the growth of the egg chamber, the general system can be written as:


$$\left\{ \begin{array}{c} \frac{\partial L(t,x)}{\partial t}+\nabla \cdot \left(v(t,x)\;L(t,x)\right)=D\;\Delta_{\text{t}}L(t,x)+V(t,x)+{ℛ}_{1}(L(t,x),P(t,x))\\ \frac{\partial P(t,x)}{\partial t}+{\nabla \cdot \left(v(t,x)\;P(t,x)\right)\;=ℛ}_{2}\left(L(t,x),P(t,x)\right)\;\;\;\;\;\;\;\;\;\;\;\;\;\;\;\;\;\;\;\;\;\;\;\;\;\;\;\;\;\;\;\;\;\;\;\;\;\;\;\;\;\;\; \end{array}\right.\;$$ (4)

5 – ***Shift of the overlying FCs***

The FCs gradually shift from cuboidal to columnar-shaped cells. During this shift, the FCs transition from overlaying the full egg chamber at S7 to engulfing only the oocyte by the end of S9 (Table 2 and S1 Fig S1 Text) [32]. Since the receptors, receptor-ligand complexes, intracellular signaling components, and inhibitors (all denoted by the general notation $\begin{array}{c} 𝑃 \end{array}$) are localized inside or on the membrane of the FCs, the posterior shift of the cells over the changing position of GRK secretion affects them. This phenomenon is transcribed mathematically by adding another transport term to the equations of these variables. We introduce a time-dependent vector field w tangent to the surface of the prolate spheroid, whose time and space-dependence will be deduced from experimental measurements.

**Table 2:** Measurements of the dimensions of the egg chamber.

Stage	S7	S8(E)	S8(L)	S9(E)	S9(L)	S10A
Time (hr)	3	7.5	10.5	13.5	16.5	19.5
$L_{E}(\mu m)$	71 (2.1)	99 (2.5)	132 (3.7)	190 (4.5)	246 (5.9)	307 (7.9)
$L_{O}(\mu m)$	–	19 (1.1)	31 (1.2)	63 (3.7)	111 (3.1)	149 (3.2)
$L_{FC}(\mu m)$	71 (2.1)	99 (2.5)	132 (3.7)	127 (5.4)	131 (4.5)	149 (3.2)
$W_{E}(\mu m)$	45 (2.1)	61 (2.9)	74 (2.8)	96 (3.2)	102 (2.8)	129 (4.3)
$W_{O}(\mu m)$	–	46 (2.7)	58 (1.8)	90 (3.7)	100 (2.6)	129 (4.3)


$$\left\{ \begin{array}{c} \frac{\partial L(t,x)}{\partial t}+\nabla \cdot \left(v(t,x)\;L(t,x)\right)=D\;\Delta_{\text{t}}L(t,x)+V(t,x)+{ℛ}_{1}(L(t,x),P(t,x))\\ \frac{\partial P(t,x)}{\partial t}+{\nabla \cdot \left(v(t,x)\;P(t,x)\right)=\nabla \cdot \left(w(t,x)\;P(t,x)\right)+ℛ}_{2}\left(L(t,x),P(t,x)\right)\; \end{array}\right.\;\;\;$$ (5)

### Detailing the reaction pathway and calibrating the model parameters

Next, we specified the interacting molecules with the ligand that produce signaling; these until now were denoted by $\begin{array}{c} 𝑃 \end{array}$. In [58], a model considering the interplay of three quantities: ligand in the extracellular space, ligand-receptor complexes, and ligand-releasing protease for cell communication in epithelial layers was built. In [60], a slightly modified version of this model is proposed with four quantities, taking into account the cell receptors in addition to the ligand, ligand-receptor complexes, and proteases. In [61], the authors design a more detailed model with six quantities: ligand is considered to be either intact or degraded; receptors are divided between surface and internalized; and ligand-receptor complexes are also divided between surface and internalized.

Building upon these models, we place ourselves at an intermediate level of complexity and focus on five quantities: ligand (L), cell receptors (R), surface ligand-receptor complexes (C), internalized ligand-receptor complexes ($C_{i}$), and signal (S). This choice is motivated by focusing on EGFR signaling and allows us to consider mechanisms of negative feedback, as well as the dynamic recycling and degradation of internalized complexes (Fig 1E). We carefully describe the evolution of the quantities that lead to EGFR signaling, including the ligand binding to the receptors, forming surface receptor-ligand complexes, which then become internalized receptor-ligand complexes, and produce the signal.

To account for negative feedback on EGFR signaling, we added the functions of the Kekkon1 (KEK1) and Sprouty (STY) inhibitors (Eq (6) and (7)). This modeling choice is new with respect to the previously cited literature and allows to monitor nonlinear effects in the production of the signal.

To include the reactions, Equation (2) for the ligand (L) is paired with ordinary differential equations for the other concentrations. Complexes C are formed by the binding of the ligand L with a receptor R, at rate ${\overset{―}{k}}_{o\text{n}}$. Conversely, $k_{\text{off}}$ gives the rate at which complexes dissociate. The complexes C at the surface of the FCs can be integrated into the cell with the rate $k_{\text{ec}}$, transforming into internalized complexes $C_{i}$. A proportion $\alpha_{\text{deg}}$ of the internalized complexes degrades at the rate $k_{\text{deg}}$, while a proportion $\alpha_{\text{rec}}$ gets recycled into surface complexes. Receptors are formed at the rate $Q_{\text{r}}$ and degrade at the rate $k_{\text{er}}$. Lastly, we consider that the signal, dpERK, is triggered due to the internalization of the ligand-receptor complexes, $C_{i}$, with the rate ${\overset{―}{k}}_{S}$. It degrades at the rate $k_{\text{d}}$. All constants are summarized in Table 1.

Omitting the time and space-dependence for concision, we can now specify the variable $P=(C,\;C_{i},\;R,\;S)$ as well as the reaction terms ${ℛ}_{1}$ and ${ℛ}_{2},\;$ and we obtain the following system of coupled partial differential equations:


$$\begin{array}{c} \left\{ \begin{array}{c} \begin{array}{c} \frac{\partial L}{\partial t}+\underset{growth}{\underbrace{\nabla \cdot (vL)}}=\underset{diffusion}{\underbrace{D\;\Delta_{\text{t}}L}}+\underset{source}{\underbrace{V}}\underset{receptor\;binding}{\underbrace{-\frac{\text{1}}{H}{\overset{―}{k}}_{o\text{n}}RL}}+\underset{unbinding}{\underbrace{k_{\text{off}}C}}\;\;\;\;\;\;\;\;\;\;\;\;\;\;\;\;\;\;\;\;\;\;\;\;\;\;\;\;\;\;\;\;\;\;\;\;\;\;\;\;\;\;\;\;\;\;\;\;\;\;\;\;\;\;\;\;\;\\ \frac{\partial C}{\partial t}+\underset{growth}{\underbrace{\nabla \cdot (vC)}}+\underset{FC\;shift}{\underbrace{\nabla \cdot (wC)}}=\underset{ligand-receptor\;binding}{\underbrace{\frac{\text{1}}{H}{\overset{―}{k}}_{o\text{n}}RL}}-\underset{unbinding}{\underbrace{k_{\text{off}}\;C}}-\;\underset{cell\;integration}{\underbrace{k_{\text{ec}}\;C}}+\underset{recycling}{\underbrace{\alpha_{\text{rec}}k_{\text{rec}}C_{i}}}\;\;\;\\ \frac{\partial C_{i}}{\partial t}+\underset{growth}{\underbrace{\nabla \cdot \left(vC_{i}\right)}}+\underset{FC\;shift}{\underbrace{\nabla \cdot \left(wC_{i}\right)}}=\underset{integration}{\underbrace{k_{ec}C}}-\underset{degradation}{\underbrace{\alpha_{\text{deg}}k_{\text{deg}}\;C_{i}}}-\underset{recycling}{\underbrace{\alpha_{\text{rec}}k_{\text{rec}}C_{i}\;}}\;\;\;\;\;\;\;\;\;\;\;\;\;\;\;\;\;\;\;\;\;\;\;\;\;\;\;\;\;\;\;\;\;\;\;\;\;\;\;\;\;\;\;\\ \frac{\partial R}{\partial t}+\underset{growth}{\underbrace{\nabla \cdot (vR)}}+\underset{FC\;shift}{\underbrace{\nabla \cdot (wR)}}=\underset{ligand\;binding}{\underbrace{\frac{\text{1}}{H}{\overset{―}{k}}_{o\text{n}}RL}}+\underset{unbinding}{\underbrace{k_{\text{off}}C}}-\underset{degradation}{\underbrace{k_{\text{er}}R}}+\underset{production}{\underbrace{Q_{\text{r}}}}\;\;\;\;\;\;\;\;\;\;\;\;\;\;\;\;\;\;\;\;\;\; \end{array}\\ \begin{array}{c} \;\\ \frac{\partial S}{\partial t}+\underset{growth}{\underbrace{\nabla \cdot (vS)}}+\underset{FC\;shift}{\underbrace{\nabla \cdot (wS)}}=\;\underset{production}{\underbrace{k_{S}C_{i}}}-\underset{degradation}{\underbrace{k_{\text{d}}S}}.\;\;\;\;\;\;\;\;\;\;\;\;\;\;\;\;\;\;\;\;\;\;\;\;\;\;\;\;\;\;\;\;\;\;\;\;\;\;\;\;\;\;\;\;\;\;\;\;\;\;\;\;\;\;\;\;\;\;\;\;\;\;\;\;\;\;\;\;\;\; \end{array} \end{array}\right.\; \end{array}$$ (6)

To represent the system more accurately, we considered the effect of negative feedback on signaling reaction rates (here, ${\overset{―}{k}}_{o\text{n}}$ and ${\overset{―}{k}}_{S}$) which will be time and space dependent. Specifically, the EGFR inhibitors, including Argos (ARG), KEK1, and STY, regulate eggshells’ patterning [30,42,46,62,63]. However, ARG is not expressed during S7 to S10A of oogenesis, hence it is not included in our model [46,64]. During S7-10A, KEK1 directly interacts with the EGF receptor to inhibit receptor dimerization. Consequently, it prevents EGFR dimerization and signaling [63]. A simple way to model this effect is to render the binding rate ${\overset{―}{k}}_{o\text{n}}$ dependent of the concentration of KEK1. On the other hand, STY acts downstream of the receptor on RAS to inhibit ERK phosphorylation [40,65]. We choose to model this effect by making the signal production rate ${\overset{―}{k}}_{s}$ dependent on STY. Thus, by adding these two inhibitors, ${\overset{―}{k}}_{o\text{n}}$ and ${\overset{―}{k}}_{s}$ become time and space-dependent, and their expression is given by:


$$\left\{ \begin{array}{c} {\overset{―}{k}}_{o\text{n}}(t,x)=\;\frac{\;k_{\text{on}}}{\text{1}+\gamma_{\text{KEK1}}\;K^{-\text{1}}\;\text{KEK1}(t,x)}\\ {\overset{―}{k}}_{s}(t,x)=\;\;\frac{\;k_{s}}{\text{1}+\gamma_{\text{STY}}\;K^{{}^{\prime }-\text{1}}\;\text{STY}(t,x)}\;\;\; \end{array}\right.\;\;$$ (7)

where the constants $k_{\text{on}}$, $k_{s}$, K and $K^{\prime }$ are given in the Table 1 and in Equations (S3) and (S4) in S1 Text.

Assuming that the production of the inhibitors depends on the signal concentration, the kinetics of STY and KEK1 are in turn given by the following equations:


$$\left\{ \begin{array}{c} \frac{\partial \;\text{STY}(t,x)}{\partial t}+\nabla \cdot (v\;\text{STY})+\nabla \cdot (w\;\text{STY})=k^{\text{STY}}S(t,x)-k_{d}^{\text{STY}}\;\text{STY}(t,x)\\ \frac{\partial \;\text{KEK1}(t,x)}{\partial t}+{\nabla \cdot (v\;\text{KEK1})+\nabla \cdot (w\;\text{KEK1})=k}^{\text{KEK1}}S(t,x)-k_{d}^{\text{KEK1}}\;\text{KEK1}(t,x) \end{array}\right.\;$$ (8)

where $k^{\text{STY}}$, $k_{d}^{\text{STY}}$, $k^{\text{KEK1}}$ and $k_{d}^{\text{KEK1}}$ are the production rates and degradation rates of STY and KEK1 (see also Section 1 in S1 Text).

The full model summarized by Equations (5–7) is purely phenomenological, hence, it needs to be tuned with data. Most parameters of the model were taken either from the literature or directly from our experimental measurements (Table 1). We calibrated the dimensions of the egg chamber, nucleus movement, and the source of ligand/GRK (S1 Fig in S1 Text). At the same time, the strengths of the inhibitors ($\gamma_{\text{STY}}$ and $\gamma_{\text{KEK1}}$, see Equation (6) are unknown.

**Fig 2:**
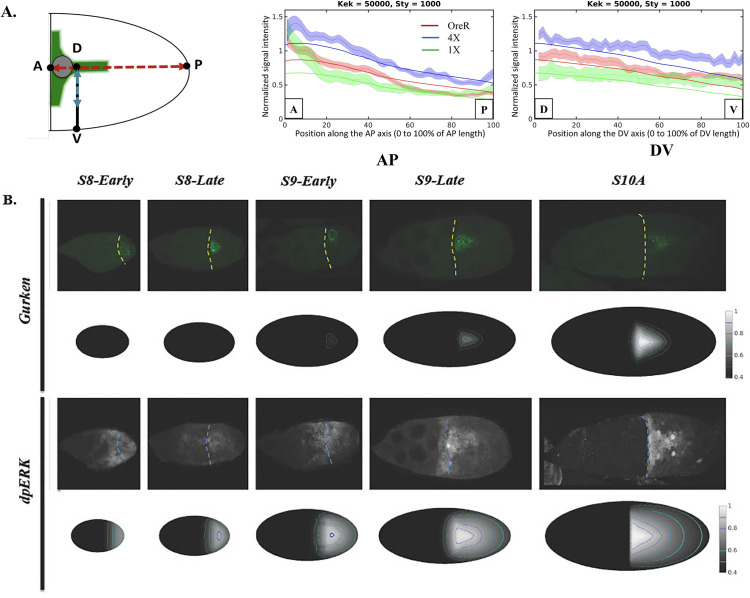
A. Images from Wild type (2x GRK, red), 4X GRK (blue), and 1x GRK (green) were measured for pixel intensities at the anterior-posterior and dorsal-ventral region of the egg chamber directly after the oocyte nucleus. These values were averaged and then plotted against simulations with many combinations of parameter values. Parameter values were selected for the best fit plots for AP and DV over the five stages considered in this model. **B.** Immunohistochemistry and model predictions for Gurken and dpERK at stages 8-early to stage 10A.

The constants $\gamma_{\text{STY}}$ and $\gamma_{\text{KEK1}}$ were then determined using data obtained from intensity plot profiles from immunostainings of GRK and dpERK at each of the modeled developmental stages (Figs 2 and S4). We reasoned, these inhibitors are induced by EGFR signaling, hence changing the levels of GRK will induce different levels of signaling at the corresponding buffering levels by the inhibitors. We used three levels of GRK corresponding to the wild-type GRK^WT^ (2 copies), 1x GRK (1 copy) and GRK^2PX^ (4 copies). Utilizing qRT-PCR, we validated that the levels of *grk* mRNA corresponds to the copy number of *grk* genes in each background. In the model, this corresponds to setting the initial flux of ligand respectively to $V_{\text{0}}$, to $\frac{\text{1}}{\text{2}}V_{\text{0}}$ and to $\text{2}V_{\text{0}}$. Experimental intensities at the AP and DV axes were compared in $L_{\text{1}}$-norm to simulation results for different values of $\gamma_{\text{STY}}$ and $\gamma_{\text{KEK1}}$. The parameters $\gamma_{\text{STY}}=\text{500}\times {\text{10}}^{\text{2}}$ and $\gamma_{\text{KEK1}}=\text{1}\text{0}\times {\text{1}\text{0}}^{\text{2}}$ showed the best fit to the experimental measurements (Fig 2A).

To summarize, we recall the three categories of parameters, classified according to the method by which we set their values.

- Parameters taken from the literature: diffusion rate (D), reaction rates ($k_{\text{ec}}$, $k_{\text{on}}$, $k_{\text{off}}$, $k_{\text{er}}$, $k_{\text{rec}}$, $k_{\text{deg}}$, $k_{\text{d}}$, $\alpha_{\text{rec}}$,$\;\alpha_{\text{deg}}$), initial flux of ligand ($V_{\text{0}}$).
- Parameters measured experimentally: physical dimensions of the egg chamber ($L_{AP}$, $L_{DV}$, $L_{O}$, $L_{FC}$, H), initial concentration of receptors ($R_{\text{0}}$), receptor production rate ($Q_{r}$), dimensions of the source of ligand.
- Parameters calibrated by comparing simulations and immunostainings of GRK and dpERK: strengths of inhibitors ($\gamma_{\text{STY}},\gamma_{\text{KEK1}})$.

Obtained by numerical simulations with the chosen parameters, our model can successfully capture the distribution of GRK/dpERK on a growing manifold with a moving morphogen source (Fig 2B). Of importance, our model is sufficient to recapitulate the dual concaved midline pattern of GRK and dpERK at S9-10A. See also Section 3 in S1 Text, for further details.

### *Using the model to determine the reduction of STY and EGFR* in genetic perturbations

**Fig 3:**
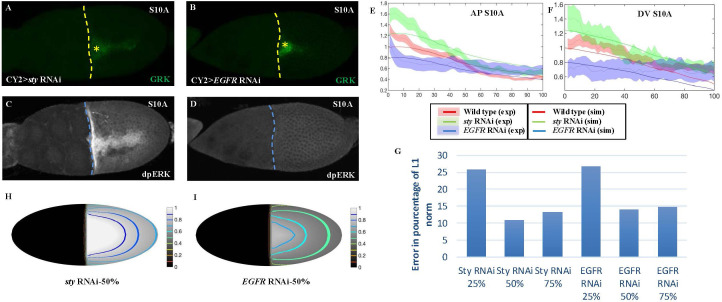
Validating parameter selections using genetic perturbations. **A-D**. Immunohistochemistry staining at S10A for **A.**GRK in Cy2 > EGFR RNAi (n = 10), **B.** GRK in *sty* RNAi (n = 11), C. dpERK in Cy2 > *EGFR* RNAi, **D.** GRK in CY2 > *sty* RNAi. **E-F**. AP and DV, respectively, dpERK intensity profiles of wild-type (red), *sty* RNAi (green) and *EGFR* RNAi (blue) and corresponding intensity profiles of simulations where RNAi targets were reduced by half. Experimental data is represented by the shaded areas representing standard deviation, and simulation results are represented by the simple curves. The wild-type simulation corresponds to the parameters defined in Tables 1 and 2. The *sty* RNAi perturbation corresponds to a modified $\gamma_{\text{sty}}(t)=\text{0}$ for all 𝐭>6, to cancel the effect of Sty on the dynamics after Stage 7. **G.** L1-relative error between dpERK experimental data represented in panels E-F and the levels predicted by the simulations, by depleting Sty (respectively EGFR) to respectively 25%, 50% and 75% of its wild-type level. **H-I.** Simulations results in 2D for a 50% Sty (respectively EGFR) depletion.

Next, we tested whether the model can be used to predict the reduction in the levels of components in the system. To test whether the *in silico* model faithfully recapitulates the *in vivo* observations, we compared the patterns of GRK and dpERK in two perturbed backgrounds. Using styRNAi to deplete *sty*, we increased the levels of dpERK (Fig 3A, 3C, and 3H). In contrast, using *egfr*RNAi, we knocked down *egfr* and reduced GRK localization as well as depleted dpERK (Fig 3B, 3D, and 3I). Note that neither perturbation affected the intensity of GRK (Figs 3A, 3B, and S4 in S1 Text).

The depletion of *sty* was modeled by reducing its production rate $k^{\text{STY}}$, whereas EGFR depletion was modeled by decreasing both the initial concentration of receptors $R_{\text{0}}$ and the receptor production rate $Q_{r}$. Qualitatively, we were able to assess the correct behavior of the model: depleting STY in the model led to a signal increase, while depleting EGFR in the model led to a signal decrease, as observed experimentally (Fig 3H and 3I). Quantitatively, the numerical simulations allowed us to estimate the amount by which *sty* and *egfr* were depleted by the RNAi, which is unknown. More specifically, several simulations were run to predict the amount of reduced *sty*, for values of its production rate $k^{\text{STY}}$ diminished to 25%, 50% and 75% of wild-type. Similarly, to estimate the effect of RNAi on *egfr* depletion, several simulations were run by setting both $R_{\text{0}}$ and $Q_{r}$ to 25%, 50% and 75% of their wild-type value (Fig 3E–3F). We then computed the error between the experimental and simulated concentrations of dpERK along the AP and DV axes at stage 10A (Fig 3E and 3F). We found that for both for STY and EGFR, the error is the smallest for a level reduced to 50% of wild-type (see Methods section). The resulting simulation results for wild-type, for a 50% depletion of sty RNAi and for a 50% depletion of EGFR RNAi are presented as solid curves along the AP axis (Fig 3E) and in the DV axis (Fig 3F), and are plotted against the experimental results (curve surrounded by shaded area) for comparison.

### Using the model to simulate perturbations of the morphological evolution

**Fig 4:**
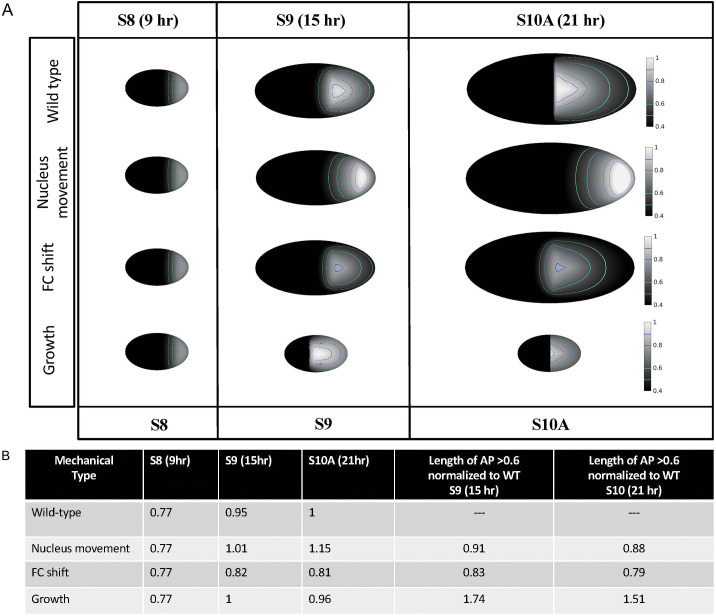
Exhibiting the role of mechanistic changes. The model was used to predict the effect of mechanistic perturbations on the distribution of dpERK. Each row shows characteristics of dpERK signal at stages 8, 9 and 10A for a different mechanistic perturbation. First row: wild-type simulations for comparison. Second row: the nucleus movement was stopped at late S8 (t = 10.5h). Third row: the follicle cell shift was removed. Fourth row: growth was stopped at lates S8 (t = 10.5h). The first three columns show the maximal value of the dpERK signal for the corresponding stage and perturbation. The fourth and fifth columns show the elongation of dpERK signal (calculated as the length of signal above 0.6) expressed in percentage of WT elongation, respectively for S9 and S10A.

Our model recapitulates the dynamic pattern of dpERK in the wild type fly, as well as in numerous perturbations, including changing the levels of the ligand, receptor, and negative feedback (Figs 2, 3 and S4). The dramatic morphological changes in egg chamber between S7-S10A [33,34] raised an intriguing question regarding the contribution of morphological changes in the tissue to the distribution of EGFR signaling. We used numerous genetic perturbations to inhibit the transition from cuboidal to columnar follicle cells by expressing Pointed (PNT) [66], E-Cadherin (E-Cad) and Cadherin 74A (Cad74A) [67]. However, the expression of PNT terminated egg chamber development at S9, likely due to a developmental check point [68], and expression of Cadherins in the anterior domain did not affect egg chamber development, these look like the wild type eggs. Hence, using the model, we explored the role of the mechanistic perturbations on the distributions of dpERK. We perturbed individually in numerical simulations the movement of the oocyte nucleus, the posterior shift of the FCs, and the growth of the egg chamber (Fig 4). We show snapshots of the egg chambers at S8, S9 and S10A with the various perturbations obtained via numerical simulations, and perform a quantitative analysis of the signaling intensity and distribution. The intensity was measured as the maximum value of dpERK over the total egg chamber. Signal elongation was computed as the length of signal above 50% of the wild type’s maximum value at the corresponding stage.

All simulations were compared to the patterns of dpERK in the wild type background at S8-10A (top row). The second row represents dpERK simulation when the nucleus remained at the posterior end at late S8 and fails to anchor to the cortex of the oocyte (t=10.5h). As expected, dpERK is found only in the posterior of the egg chamber (Fig 4A). In the third row, we numerically stopped the transition from cuboidal to columnar FCs. This numerical perturbation does not have any effect on the maximum signal intensity but affects the signal length at S10A: without the shift of the FCs, the signal elongation (calculated as the AP length of the surface with dpERK above 0.6) increases from 278 to 302 micrometers (Fig 4B), which is a 9% increase. Interestingly, the elongation is mostly observed towards the anterior side of the egg chamber at S10A.

The fourth row of Fig 4A shows the pattern of dpERK when growth of the egg chamber is numerically stopped at late S8 (t=10.5h). This mechanistic change has two consequences. First, it affects the diffusion of the signal as the growth affects the curvature of the perivitelline space, which in turn affects the Laplace-Beltrami operator. At earlier stages, the egg chamber’s A/P and D/V dimensions are comparable, so the egg chamber is almost spherical. Consequently, diffusion at earlier stages is almost isotropic. At later stages, wild-type egg chambers are more elongated, so this earlier symmetry is broken, and diffusion becomes anisotropic. This biased diffusion of GRK affects the shape of the signal. Secondly, the numerical perturbation of growth also affects the signal intensity, i.e., as the egg chamber remains of small size, the source intensity is lower, which leads to lower levels of dpERK. The lower levels of GRK in our model is because the total amount of GRK secretion is proportional to the total surface of the oocyte (S2 Fig and Equation (S6) in S1 Text).

## Discussion

Our model provides a novel platform to represent cell signaling over evolving morphology with dynamic source of a ligand. We bring new insights into the contributing mechanisms to the distribution of GRK and EGFR signaling. First, our experimental choice, *Drosophila* oogenesis, facilitated the determination of values for parameters that have so far only been measurable in culture cells [58]. These parameter values were then applied to a highly-dynamic model that takes into account the mechanistic features of the egg chamber, its overall growth, the shifting epithelial cells, and a moving morphogen source. These features are in contrast to current models that simulate the distribution of GRK/dpERK at the final steady-state stage [48,49]. We provide a new approach by building output from earlier stages to achieve a continuous dynamic evolution of signaling distribution over time (S7-10A). To accomplish accuracy, we took careful measurements of all egg chamber compartments and the source of GRK. We found that unlike the previous use of a dome-like shaped GRK source [48], GRK is distributed in a “T” shaped pattern already while in the oocyte (S2 Fig in S1 Text).

Observations of EGFR dynamics and overall growth made it necessary to piece apart stages 8 and 9 into additional stages, early and late. Furthermore, we were able to use values in current literature and build on previous models for the dynamics of the EGFR, GRK diffusion, internalization of GRK/EGFR complexes, and FC counts [48,49,55,58,61,69]. The unknown parameters were approximated by running numerous simulations against 1-D intensity plots. Interestingly, the dorsal anterior-most points of the experimental 1-D plots recorded higher intensity values than the 1-D simulations (Fig 3E). This discrepancy can be explained by the activation of EGFR (dpERK) in the border cells, a group of 6–8 epithelial cells delaminated from the anterior and migrating during S9 through the nurse cells to end up in the proximity of the oocyte nucleus at the dorsal anterior outside the oocyte [31,33]. We note that we focused on oocyte-FCs interactions, hence, the border cells are not considered in our model. Furthermore, experimental intensity values for 30% of the DV were used for comparison due to the changes in intensity detected at the outer 20% of the DV, or the edges, as an image artifact that could not be reliably captured by the model. After establishing parameters based on AP and DV experimental profiles, we were able to simulate the distribution of dpERK on a growing manifold.

Based on the levels of EGFR activation, we found that the intensity of GRK distribution predicted by the model was much higher than the experimental intensity plot profiles (Fig 2B). This could be explained by the diffusive nature of the ligand; much of the unbound GRK in the PVS is lost during egg chambers’ fixation. This phenomenon was previously reported for a modified protocol for GRK analyses [70]. In addition, our model discovered a lower concentration of GRK in the anterior ventral FCs (not shown), which is consistent with past experimental findings [48,71–73].

Selected parameters were tested against genetic knock-down of *sty* and *egfr* by RNAi. The strength and efficiency of the RNAi are unknown as these depend on the combination between the GAL4 driver (see material and methods) and the efficiency of the tissue specific action of the RNAi. Using our model, we simulated the full reduction of dpERK when reducing the amount of receptors by half. In addition, our model associated the increased levels of EGFR signaling in the *sty*RNAi background to be at 50% reduction in STY. The predictive capabilities of the model can provide quantitative measurements for these genetic perturbations in the absence of direct molecular analyses of EGFR and STY.

After validating the performance of the model, we used its predictive capabilities to determine how the changes in size and morphology of egg chamber’s compartments, including morphogen source, FCs columnar shift, and overall growth, contribute to the distribution of dpERK. Stopping nuclear movement at late S8, the model shows a high concentration of dpERK at the posterior end of the egg chamber. Interestingly, at S10A, the signal has a higher intensity than wild-type S10A above the nucleus, and its elongation is also greater. The high intensity above the nucleus is explained by the fact that the posterior has been exposed to the source of ligand continuously from S7 to S10A, whereas in the wild-type, the region above the nucleus (the dorsal anterior of the oocyte at S10A) has been exposed to the source of ligand for a much shorter time (S2 Fig in S1 Text). This highlights the important role of nuclear movement in creating a comet-like trace of GRK along the dorsal most side of the egg chamber. With a moving nucleus, the model shows that the points towards the posterior of the nucleus retain a memory of its passage, and thus have a comparatively higher intensity than points at the same distance, but not having been exposed to the source of ligand. As a result, the intensity gradient from anterior to posterior is much steeper without nucleus movement than in wild-type.

These results are similar to the previously shown treatment with colchicine, an agent that depolymerizes microtubules in the oocyte, causing the nucleus to remain at posterior end [53]. However, the disruption of microtubules also disrupts the localization of *grk* mRNA [74], and consequently lowers the level of GRK protein and the activation of EGFR. Our model does not take into consideration the microtubules, hence the perturbation recapitulates dpERK under circumstances where *grk* mRNA localization is maintained near the oocyte nucleus at the posterior, which is the place of GRK translation.

Remarkably, revoking the shift of the FCs has no effect on the maximum value of dpERK in our model. At the same time, the activation of EGFR has now a longer pattern when compared to the pattern found in the wild type background. This perturbation allowed GRK to diffuse anteriorly and signal in the FCs over the nurse cells. In the wild type, these cells are stretched tidily around the nurse cells, which likely blocks GRK diffusion to the anterior cells, thus maintaining the direction of GRK diffusion to posterior and lateral domains in reference to the oocyte nucleus position.

When growth is perturbed, we observe that the signal profile is less elongated, both at S9 and S10A, likely due to the size of the oocyte. However, when normalized to the total circumference of the oocyte in the AP direction, we found that in the wild type, the signal extension at S10A is 39% (278/711) of the total oocyte circumference. When growth is perturbed, the signal extension is 34% (116/335) of the oocyte circumference. This suggests that growth might also play a role in the signal’s extension.

Our model successfully recapitulated the spatiotemporal evolution of GRK/dpERK patterning in egg chambers. This was achieved by developing a suitable mathematical framework for reaction-diffusion equations in developing organisms. In this work, the growth of the egg chamber is a morphological change that affects the distribution of GRK through the time-evolving Laplace-Beltrami operator. This novel framework has potential applications in other growing developing tissues, when the source of the signaling ligands and tissue compartments are known. In addition, this model will be tested for its ability to simulate the diversity of GRK/dpERK distribution along the DV axis of egg chambers from species that have a dorsal ridge, a lumen-like structure, along their dorsal most portion of the eggshells [53,75].

## Materials and methods

### Fly species and stocks

The selected fly strains used were: wild type *D. melanogaster* (25211), CY2-GAL4 (Queenan *et al*., 1997), *grk*^2px^ [37], UAS-*sty* RNAi (y[1] sc[*] v[1] sev [21]; P{y[+t7.7] v[+t1.8]=TRiP.HMS01599}{Queenan, 1997 #59}attP2, BSID #36709 [76], UAS-EGFR RNAi (y[1] v[1]; P{y[+t7.7] v[+t1.8]=TRiP.JF02283}attP2, BSID#36770 [76], *grk* [2b]b, *grk* [2E12]b (gifts from Trudi Schüpbach, Howard Hughes Medical Institute, Princeton University, Princeton, NJ, USA), X7;28.20 [44]. All crosses and fly stocks were grown on cornmeal agar and maintained at 23°C.

### Immunofluorescence and microscopy

Immunoassays for dpERK were performed as previously described (Zartman *et al.*, 2009). Ovaries were mounted in Flouromount-G from Southern Biotech. Primary antibodies used were rabbit anti-dpERK (1:100, Cell Signaling Technologies #9101) diluted 1:100, and preabsorbed mouse anti-GRK (1:10, DSHB #1D12). Secondary antibodies used at a 1:2000 dilution were: Alexa Fluor 488 nm (anti-mouse, Molecular Probes #A-21202), 647 nm (anti-mouse, Molecular Probes #A-31571), and 568 nm (anti-rabbit, Molecular Probes #A-21206). Nuclear staining was performed using DAPI (84 ng/ml, Thermofisher #D1306). All immunofluorescent images were captured with a Leica SP8 confocal microscope in photon counting mode (Rutgers University, Camden Imaging Core Facility). Image acquisition was performed by using the same parameters for each of the channels for all documented egg chambers.

### Intensity profile quantification

Anterior-posterior (AP) and dorso-ventral (DV) intensities of GRK and dpERK were measured in *D. melanogaster* and batched per developmental stage. Developmental stages were grouped by stages 7, 8 (early), 8 (late), 9 (early), 9 (late), and 10A. Early and late stages 8 and 9 were determined by measurement (S1B Fig in S1 Text). Image analysis was performed in Matlab as follows: The intensities of dpERK signaling along the AP axis were measured at 100 individual points evenly distributed throughout the length of the dorsal midline, from the anterior of the columnar FCs to the posterior edge of the FCs. For stages 7 and 8, where all cells are cuboidal, intensities were measured at 100 individual points along the length of the egg chamber. The DV intensities were measured along the width of the egg chamber at 50 evenly distributed individual points. The optimal position to measure the width of GRK, where the intensities were extracted along the DV, is the posterior of the oocyte nucleus. To circumvent autofluorescence along the edges of the egg chamber, the 50 points collected from the DV measurements were taken from 30% of the egg chamber beginning at the very center (S2 Fig in S1 Text). Within each stage, intensities were among a minimum of fifteen images from three independent dissections and the standard error was calculated for each of the position (point) for AP and DV.

The AP and DV experimental intensities of GRK and dpERK were compared to numerical results by extracting from the simulations the intensity of dpERK over the AP or DV lines at simulation times corresponding to each of the development stages. The resulting curves obtained in arbitrary units (A.U.) were then superimposed to the experimental ones by normalizing them by a unique factor, chosen so that the simulation curve for wild-type AP at stage 10A matches the experimental curve at 20% of the total length (see Fig 2A).

### Numerical simulations

The complete model (5)-(7) was nondimensionalized in order to work with variables of order 1 in the numerical simulations. The nondimensionalized equations are presented in S1 Text.

The complete model that we are studying is composed of several components that pose numerical challenges in specific ways. Solving the system of partial differential equations numerically requires finding a suitable spatial discretization of the domain, in this case a two-dimensional prolate spheroid. The most natural parameterization of such a symmetric surface would be the prolate spheroidal coordinates as used previously in [48]. However, this system of coordinates is degenerate at the two poles of the spheroid. In consequence, the corresponding mesh constructed with prolate spheroidal coordinates is ill-suited for the numerical approximation of diffusion. As an alternative to prolate spheroidal coordinates, we used cubed spheroidal coordinates, adapted from the cubed sphere coordinates developed in [77] and [78]. More specifically, the quarter prolate spheroid is divided into 4 zones, each parameterized by a separate set of coordinates $\left(x_{i},y_{i}\right)$, with i∈[0,…,3]. Matching boundary conditions are set at the interfaces between zones. Neumann boundary conditions, provided by symmetry considerations and zero-flux conditions are set at the boundary of the quarter prolate spheroid. The other quarter prolate spheroid is obtained by symmetry (S3 Fig in S1 Text).

We then solved the system of coupled PDEs (5)-(6)-(7) by operator splitting, using the forward Euler method. Each time step is further divided into four sub-steps in which we treat independently:

- diffusion: the time-varying Laplace-Beltrami operator is approximated with finite differences in each zone;
- growth: all variables are transported via the vector field v to the new prolate spheroid;
- shift of follicle cells: all variables except the ligand L are transported towards the oocyte posterior via the vector field w;
- reactions: the system is now reduced to coupled ODEs

All computations and simulations were done in MATLAB. For details, see Section 2 in S1 Text.

### Error estimation

To compare the experimental measurements of dpERK intensity signaling at S10A with the simulation results of dpERK concentration at S10A, both were plotted along the AP axis (Fig 3E) and DV axis (Fig 3F). For the experiment consisting in deleting Sty by Sty RNAi (green shaded curve in Fig 3E and 3F), three simulation curves were obtained respectively by depleting STY to 25%, 50% and 75% of its wild-type level. The relative error between the experimental curves and each of these simulation curves was computed in L1 relative error, by computing the integral of the absolute value of the difference between the two curves and dividing it by the integral of the experimental curve. The sums of AP and DV errors are represented in Fig 3G. The best-fitting simulation curve, corresponding to 50% depletion by the *sty*RNAi, is represented in Fig 3E and 3F (green line), along with the experimental curve. The same protocol was applied to EGFR depletion by *egfr*RNAi (in blue in Fig 3E and 3F). The corresponding 2D simulations are represented in Fig 3H and 3I.

### qPCR and RNAi validation analysis

RNA was extracted from egg chambers at stages of mid-oogenesis (S9-S11). Briefly, sixty egg chambers were collected from each genetic background: (CY2 > *sty*RNAi, CY2 > *egfr*RNAi, wild type *D. melanogaster OreR*) into 300 µL TriReagent (Zymo Cat: R2050-1–50). Samples were collected in biological duplicates and homogenized using a dounce homogenizer. RNA was extracted with **Quick**-RNA mini-prep kit (Zymo Cat: R1054). RNA was treated with DNase I (0.08 U/µL, NEB, M0303), Exonuclease I (0.8 U/µL NEB, M0293), and Exonuclease III (4U/µL, NEB, M0206) to remove genomic DNA contamination. RT was performed with random hexamers using NEB protoscript II kit (E6560) and followed manufacturer’s instructions with 1 µg total RNA per reaction. No RT controls were performed by adding 50 ng RNA directly to 384-well plate. The experiment was run in two biological replicated and repeated twice. Samples were normalized to the RPL32-RA as previously described [79]. The qPCR analysis was performed using PowerUp SYBR green (Applied Biosystems, A25780) and run at manufacturer’s conditions on a Quant Studio 6 Flex Real-Time PCR system. Primers were designed against the 5’ transcript according to previous findings in [80]. Fold change was calculated by deducting the mean RPL32 cycle threshold (Ct) from each technical replicate (OreR and perturbation) and taking the mean of all biological and technical replicates. Fold change was computed by LOG10 [*OreR* Ct/genetic perturbation Ct].

## Supporting information

S1 TextS1 Fig. Dynamic measurements of the egg chamber dimensions from S7 to S10A.Data was collected at 6 consecutive time-points: 3hr (S7), 7.5hr (S8E), 10.5hr (S8L), 13.5hr (S9E), 16.5hr (S9L), 19.5hr (S10AE). A. Three measurements were taken in the AP direction: the total length of the egg chamber 𝐿_𝐸_, the length of the oocyte 𝐿_0_, and the length of the follicle cells 𝐿_𝐹C_. **B.** A cartoon schematic showing positions of measurements along the AP axis. **C.** Two measurements were taken in the DV direction: the egg chamber width 𝑊_𝐸_ and the oocyte width near the oocyte nucleus 𝑊_0_. **D.** A cartoon schematic showing positions of measurements along the DV axis. Note that the semi-axes of the prolate spheroid modeling the oocyte then correspond to half of the egg chamber length and width: $L_{AP}:=\frac{LE}{\text{2}}$ and $L_{DV}:=\frac{WE}{\text{2}}$ **E.** A cartoon schematic showing the different morphological transformations taken into account. The growth vector field, perpendicular to the egg chamber surface, is schematized by blue arrows. The follicle cells’ shift, tangent to the egg chamber surface, is represented by green arrows. The oocyte nucleus’ movement from (P) to (D), in the plane (xOz) is indicated by a grey arrow. **S2 Fig.** Measurements of the source. **A.** A cartoon depicting the vantage points from where source measurements were taken at the (i) Sagital, denoted by red dotted line, (ii) Dorsal, denoted by yellow dotted line, and (iii) Anterior, denoted by blue dotted line. **B.** Immunohistochemistry stainings from vantage points of the egg chamber at S10A: (i) Sagital (ii) Dorsal measurement from a ventral view (iii) Anterior boundary of oocyte. Corresponding table provides ratios of length of source compared to total length of domain (i.e. Sagital (Sag), Dorsal (D), Anterior (A)) at S8 (n=6 D,A), S9 (n=8 for D, Sag,; n =10 A), and S10A (n=5 for D,Sag, n=9 for A). More precisely, Ratio (i) corresponds to the ratio of the dorsal length of the source with respect to the total (curved) length of the oocyte. Ratio (ii) corresponds to the ratio of the width of the source at the posterior of the nucleus with respect to the width of the oocyte at the posterior of the nucleus. Ratio (iii) corresponds to the ratio between the curved width of the signal at the anterior of the nucleus and the half perimeter of the oocyte at the anterior of the nucleus. **C.** Representation of the numerical implementation of the source and table of experimental measurements of the source at specific stages. At S10A we show the current numerical implementation of the source compared to the source used in (Goentoro et al., 2006). **S3 Fig. A.** Parametrization of the prolate spheroid representing the egg-chamber at a given time *t* by ^(^𝜂, 𝜃^)^ ∈ ^[^0, 𝜋^]^ × [0,2𝜋]. The lengths of its semi-axes are 𝐿_𝐴𝑃_(𝑡) (along the z- axis), and 𝐿_𝐷𝑉_(𝑡) (along the x and y-axes). The posterior (𝑃) corresponds to 𝜂 = 0, and the anterior (𝐴) to 𝜂 = 𝜋. The dorsal side (𝐷) corresponds to 𝜃 = 0 and the ventral side (𝑉) to 𝜃 = 𝜋. The oocyte nucleus, represented by the gray circle, migrates from the posterior to the dorsal anterior of the spheroid. **B-E**. Construction of the cubed spheroidal mesh. The cubed spheroidal mesh is obtained by a two-step transformation, from the cube 𝐶_𝑎_ to the sphere 𝑆_𝐴𝑃_, and from the sphere 𝑆_𝐴𝑃_ to the spheroid 𝑃. Consider the sphere of radius 𝑆_𝐴𝑃_ centered at 0 and let 𝐶_𝑎_ be its inscribed cube of side $\text{2}a=\text{2}\frac{LAP}{\sqrt{\text{3}}}$, also centered at 0. Each side of 𝐶_𝑎_ is discretized by a regular orthonormal mesh (**D**). Then the cubed spherical mesh of 𝑆_𝐴𝑃_ is obtained by taking the radial projection of the mesh of 𝐶_𝑎_ onto 𝑆_𝐴𝑃_ (**D** and **B**): each vertex 𝑃_C_^(^𝑥_𝑐_, 𝑦_𝑐_, 𝑧_𝑐_^)^ ∈ 𝐶_𝑎_ of the mesh is projected radially onto 𝑆_𝐴𝑃_, giving the point 𝑃_𝑆_^(^𝑥_𝑆_, 𝑦_𝑆_, 𝑧_𝑆_^)^ ∈ 𝑆_𝐴𝑃_. By this transformation, the sphere 𝑆_𝐴𝑃_ is meshed by the cubed sphere projection. Secondly, each vertex 𝑃_𝑆_ ∈ 𝑆_𝐴𝑃_ of the cubed spherical mesh is projected onto the prolate spheroid 𝑃, orthogonally to the 𝑧-axis (**C**). This transformation defines the cubed spheroidal mesh (**E**). **F-G.** Division of the prolate spheroid 𝑃 into subdomains. From the cubed spheroid mesh construction, each quarter spheroid is divided into 4 subdomains, corresponding to four sides of the cube. In S3 Fig F, we represent the quarter spheroid contained in the region ^{(^x, y, z^)^ ∈ ℝ^3^, 𝑦 ≥ 0, 𝑧 ≥ 0^}^. It is divided into the subdomains 𝑆_0_ (covering the posterior pole), 𝑆_1_ (dorsal region), 𝑆_2_ (lateral region) and 𝑆_3_ (ventral region). S3 Fig G shows the boundary conditions implemented at the subdomain interfaces. Dotted lines represent matching boundary conditions, whereas continuous lines indicate Neumann boundary conditions (due to the symmetry property). The mesh for the region ^{(^x, y, z^)^ ∈ ℝ^3^, 𝑦 ≤ 0, 𝑧 ≥ 0^}^ is obtained by symmetry with respect to the plane XZ, and the meshes for the regions ^{(^x, y, z^)^ ∈ ℝ^3^, 𝑦 ≥ 0, 𝑧 ≤ 0^}^ and ^{(^x, y, z^)^ ∈ ℝ^3^, 𝑦 ≤ 0, 𝑧 ≤ 0^}^ are then obtained by symmetry with respect to the plane XY. In fact, the problem is symmetric with respect to the plane (XZ), so the solution is computed only for the semi-spheroid 𝑦 ≥ 0.**S4 Fig.** Intensity plots of dpERK and GRK at stages 8 through 10A of oogenesis. **A.** Intensity profiles of GRK for varying GRK copy numbers at 2x (OreR), 4x, and 1x along the AP (top row) and DV (bottom row). **B.** Intensity profiles of dpERK for varying GRK copy numbers at 2x (OreR), 4x, and 1x along the AP (top row) and DV (bottom row). **C.** Intensity profiles of GRK for three genetic backgrounds: wild-type (OreR), STY- RNAi, and EGFR-RNAi along the AP (top row) and DV (bottom row). **D.** Intensity profiles of dpERK for three genetic backgrounds: wild-type (OreR), STY- RNAi, and EGFR-RNAi along the AP (top row) and DV (bottom row).(PDF)
