# Supplementary material for: A computational framework to study EGFR signaling distribution in egg chambers during dynamic interactions between soma and germline
Source: PLoS Comput Biol. 2025 Dec 29;21(12):e1013802. doi: 10.1371/journal.pcbi.1013802 (PMC12826490; doi:10.1371/journal.pcbi.1013802)
Supplement: S1 Text — Data was collected at 6 consecutive time-points: 3hr (S7), 7.5hr (S8E), 10.5hr (S8L), 13.5hr (S9E), 16.5hr (S9L), 19.5hr (S10AE). A. Three measurements were taken in the AP direction: the total length of the egg chamber 𝐿𝐸, the length of the oocyte 𝐿0, and the length of the follicle cells 𝐿𝐹C. B. A cartoon schematic showing positions of measurements along the AP axis. C. Two measurements were taken in the DV direction: the egg chamber width 𝑊𝐸 and the oocyte width near the oocyte nucleus 𝑊0. D. A cartoon schematic showing positions of measurements along the DV axis. Note that the semi-axes of the prolate spheroid modeling the oocyte then correspond to half of the egg chamber length and width: LAP:=LE2 and LDV:=WE2 E. A cartoon schematic showing the different morphological transformations taken into account. The growth vector field, perpendicular to the egg chamber surface, is schematized by blue arrows. The follicle cells’ shift, tangent to the egg chamber surface, is represented by green arrows. The oocyte nucleus’ movement from (P) to (D), in the plane (xOz) is indicated by a grey arrow. S2 Fig. Measurements of the source. A. A cartoon depicting the vantage points from where source measurements were taken at the (i) Sagital, denoted by red dotted line, (ii) Dorsal, denoted by yellow dotted line, and (iii) Anterior, denoted by blue dotted line. B. Immunohistochemistry stainings from vantage points of the egg chamber at S10A: (i) Sagital (ii) Dorsal measurement from a ventral view (iii) Anterior boundary of oocyte. Corresponding table provides ratios of length of source compared to total length of domain (i.e. Sagital (Sag), Dorsal (D), Anterior (A)) at S8 (n=6 D,A), S9 (n=8 for D, Sag,; n =10 A), and S10A (n=5 for D,Sag, n=9 for A). More precisely, Ratio (i) corresponds to the ratio of the dorsal length of the source with respect to the total (curved) length of the oocyte. Ratio (ii) corresponds to the ratio of the width of the source at the posterior of the nucleu [file pcbi.1013802.s001.pdf]

Supplemental Material

Section 1: Model Construction

1.1. The construction of the phenomenological model

A- Reactions between ligand, receptors, complexes, inhibitors and signal:

The flow of ligand from the oocyte nucleus is denoted by V. The ligand binds to the receptors with the binding rate $\bar{k}_{on}$ and dissociation rate k_{off} (Fig 1D,1E). The receptors are produced at a rate Q_{r} and degraded at a rate k_{er}. The ligand-receptor complex is then internalized at the rate k_{ec}, and subsequently the internalized complex can be degraded at rate k_{deg} or recycled at rate k_{rec}. The internalized complex activates the EGFR signaling pathway in the overlaying follicle cells, at the rate $\bar{k}_S$. The output of the Ras-Raf-MAPK signaling cascade, dpERK, regulates the transcription of genes by controlling the phosphorylation state of transcription factors, for our model, regulators of the pathway (i.e. inhibitors). It degrades at the rate k_{d}.

All variables are functions of time t and of the two space coordinates (η, θ) that parameterize the surface (S3 Fig A). The system of reaction-diffusion equations can be written as:

$$\begin{cases} \dfrac{\partial L}{\partial t} + \nabla \cdot (vL) = D\,\Delta_{\text{t}} L + V - \dfrac{1}{H}\bar{k}_{\text{on}} RL + k_{\text{off}} C \\ \dfrac{\partial C}{\partial t} + \nabla \cdot (vC) + \nabla \cdot (wC) = \dfrac{1}{H}\bar{k}_{\text{on}} RL - (k_{\text{off}} + k_{\text{ec}})\, C + \alpha_{\text{rec}} k_{\text{rec}} C_i \\ \dfrac{\partial C_i}{\partial t} + \nabla \cdot (vC_i) + \nabla \cdot (wC_i) = k_{ec} C - \alpha_{\text{deg}} k_{\text{deg}}\, C_i - \alpha_{\text{rec}} k_{\text{rec}} C_i \\ \dfrac{\partial R}{\partial t} + \nabla \cdot (vR) + \nabla \cdot (wR) = -\dfrac{1}{H}\bar{k}_{on} RL + k_{\text{off}} C - k_{\text{er}} R + Q_{\text{r}} \\ \dfrac{\partial S}{\partial t} + \nabla \cdot (vS) + \nabla \cdot (wS) = \bar{k}_S C_i - k_{\text{d}} S \end{cases} \qquad (S1)$$

where $L = L(t, \eta, \theta)$ represents the concentration of ligand (GRK), $C = C(t, \eta, \theta)$ is the concentration of surface ligand-receptor complexes, $C_i = C_i(t, \eta, \theta)$ is the concentration of internalized ligand-receptor complexes, $R = R(t, \eta, \theta)$ is the concentration of surface receptors (EGFR) and $S = S(t, \eta, \theta)$ is the concentration of signal (dpERK).

All parameters of the model are either taken from literature or measured experimentally (Table 1). The flux of ligand $V(t, \eta, \theta)$ is a function of time and space and determined by the observed shape and position of the source of ligand (S2 Fig).

At this level of model construction, the last four equations of (S1) are ordinary differential equations (briefly ODE), so we have a coupled system of four ODE and one PDE. Indeed the evolution of the surface complexes, internalized complexes, receptors and signal are so far described by pointwise dynamics. On the other hand, the evolution of ligand is modeled by a reaction-diffusion equation.

B- Inhibitors dynamics:

The kinetics of Sprouty (STY) and Kekkon1 (KEK1) are given by the following equations:

$$\begin{cases} \dfrac{\partial \text{STY}}{dt} + \nabla \cdot (v \ \text{STY}) + \nabla \cdot (w \ \text{STY}) = \ k^{\text{STY}} S - k_d^{\text{STY}} \ \text{STY} \\ \dfrac{\partial \text{KEK1}}{dt} + \nabla \cdot (v \ \text{KEK1}) + \nabla \cdot (w \ \text{KEK1}) = k^{\text{KEK1}} S - k_d^{\text{KEK1}} \ \text{KEK1} \end{cases} \quad (S2)$$

where k^{STY}, k_d^{STY}, k^{KEK1} and k_d^{KEK1} are the production rates and degradation rates of STY and KEK1. To simplify, we consider that these rates are comparable to EGFR's recycling and degradation rates (i.e. k^{STY}=k_d^{STY}=k^{KEK1}=k_d^{KEK1}=k_d).

KEK1 targets EGFR dimerization, thus reducing GRK uptake and leaving higher levels of free ligand. To account for this effect, we modify the receptor-ligand binding rate via Michaelis-Menten kinetics, as follows:

$$\bar{k}_{\text{on}}(t, \eta, \theta) = \frac{k_{\text{on}}}{1 + \gamma_{\text{KEK1}} \ K^{-1} \ \text{KEK1}(t, \eta, \theta)} \qquad (S3)$$

The parameter γ_{Kek} is the strength of inhibitory feedback of KEK1, and the constant K, defined by $K = Vk_S k^{\text{KEK1}}/(k_d k_{\text{deg}} k_d^{\text{KEK1}})$, is the concentration of KEK1 at steady state. The parameter k_{on} is the constant (in time and in space) binding rate in the absence of KEK. The modified binding rate $\bar{k}_{on}$ is a space-time-dependent variable, affected by KEK1. In the absence of KEK1, $\bar{k}_{\text{on}} \equiv k_{\text{on}}$, we recover the constant binding rate classically used in [1] and [2], see also Table 1.

STY acts on the intracellular components, affecting signal propagation (Fig 1). We include STY's negative feedback network motif in the ODE for dpERK, also via Michaelis-Menten kinetics, with:

$$\bar{k}_s(t, \eta, \theta) = \frac{k_s}{1 + \gamma_{\text{STY}} \ K'^{-1} \ \text{STY}(t, \eta, \theta)} \qquad (S4)$$

where γ_{Sty} is the strength of inhibitory feedback, $K' = Vk_S k^{\text{STY}}/(k_d k_{\text{deg}} k_d^{\text{STY}})$ is the concentration of Sty at steady state and k_s is the constant rate of activation of dpERK in the absence of STY.

C- Ligand-Receptor Complex recycling:

As represented in the system of reactions, a fraction $\alpha_{\text{deg}} \in [0,1]$ of the internalized complexes goes to degradation, while the fraction $\alpha_{\text{rec}} = 1 - \alpha_{\text{deg}}$ is recycled and goes back to the membranes of the follicle cells to be reused. To model the amount of available receptor, we consider a previous study that reported two different pathways for the EGFR internalization: clathrin-regulated endocytosis (CME) and non-clathrin-mediated endocytosis (NCE) [3]. This study found in culture cells that while 70% of the receptors internalized through the CME pathway are recycled, only 15% of the receptors undergoing NCE are recycled [3]. Importantly, at low level of ligand, almost all receptors undergo clathrin-mediated endocytosis. At high level of ligand, 60% of EGFR undergo CME and 40% undergo NCE. We incorporate this data in our model, considering that the fractions of degraded and recycled receptors depend on the level

of ligand. At low ligand, $\alpha_{\text{deg}} = 0.3$ and at high ligand, $\alpha_{\text{deg}} = 0.55$. At intermediate levels of ligand, we interpolate linearly as follows:

$$\begin{cases} \alpha_{\text{deg}}(t,\eta,\theta) = 0.3 + 0.25\dfrac{L(t,\eta,\theta) - L_{\min}(t)}{L_{\max}(t) - L_{\min}(t)} \\ \alpha_{\text{rec}}(t,\eta,\theta) = 1 - \alpha_{\text{deg}}(t,\eta,\theta) \end{cases} \qquad (S5)$$

where $L_{\min}(t)$ and $L_{\max}(t)$ are respectively the minimum and maximum values of L at time t.

D- Initial conditions for the complete model

This set of equations is supplemented by initial conditions for each of the concentrations. All concentrations are initially considered to be zero, except for the initial concentration of receptors, R_0:

$$L(0,\eta,\theta) = 0,\ \ C(0,\eta,\theta) = 0,\ \ C_i(0,\eta,\theta) = 0,\qquad R(0,\eta,\theta) = R_0,$$
$$S(0,\eta,\theta) = 0,\qquad \text{KEK1}(0,\eta,\theta) = 0,\qquad \text{STY}(0,\eta,\theta) = 0.$$

1.2. Nondimensionalizing the model

We rescale Equations (S1)-(S2) by the quantities $L_0 = \frac{HV_0}{k_{\text{I}}}$, $C_0 = \frac{V_0}{k_{\text{ec}}}$, R_0 and $S_0 = \frac{V_0}{k_{\text{d}}}$. The constant k_{I} defined by $k_{\text{I}} = \frac{k_{\text{ec}}k_{\text{on}R}}{k_{\text{off}}+k_{\text{ec}}}$ is related to the rate of internalization of ligand at steady-state, and its fundamental role in determining the shape of the signal at steady-state was discussed in [5].

Rescaling by L_0, C_0, R_0 and S_0 renders the variables dimensionless. It also ensures that the new variables $l = L/L_0$, $c = C/C_0$, $c_i = C_i/C_0$, $r = R/R_0$, $s = S/S_0$, $\text{sty} = \text{STY}/S_0$ and $\text{kek1} = KEK1/S_0$ are of the order of 1, which allows greater numerical precision. We rewrite the system of equations (S1)-(S2) in terms of the dimensionless distributions l, c, c_i, r and s:

$$\begin{cases} \dfrac{\partial l}{\partial t} + \nabla\cdot(vl) = D\,\Delta_{\text{t}} l + \dfrac{V}{L_0} - \dfrac{R_0}{H}\bar{k}_{\text{on}} rl + \dfrac{k_{\text{off}}C_0}{L_0} c \\ \dfrac{\partial c}{\partial t} + \nabla\cdot(vc) + \nabla\cdot(wc) = \dfrac{R_0 L_0}{HC_0}\bar{k}_{\text{on}} rl - (k_{\text{off}} + k_{\text{ec}})\, c + \alpha_{\text{rec}} k_{\text{rec}} c_i \\ \dfrac{\partial c_i}{\partial t} + \nabla\cdot(vc_i) + \nabla\cdot(wc_i) = k_{ec} c - \alpha_{\text{deg}} k_{\text{deg}}\, c_i - \alpha_{\text{rec}} k_{\text{rec}} c_i \\ \dfrac{\partial r}{\partial t} + \nabla\cdot(vr) + \nabla\cdot(wr) = -\dfrac{L_0}{H}\bar{k}_{on} rl + \dfrac{k_{\text{off}}C_0}{R_0} c - k_{\text{er}} R_0 r + \dfrac{Q_{\text{r}}}{R_0} \\ \dfrac{\partial s}{\partial t} + \nabla\cdot(vs) + \nabla\cdot(ws) = \bar{k}_S \dfrac{C_0}{S_0} c_{[illegible]} - k_{\text{d}} s \end{cases}$$

$$\begin{cases} \dfrac{\partial \text{sty}}{dt} + \nabla\cdot(v\ \text{sty}) + \nabla\cdot(w\ \text{sty}) = k^{\text{STY}} s - k_d^{\text{STY}}\ \text{sty} \\ \dfrac{\partial \text{kek1}}{dt} + \nabla\cdot(v\ \text{kek1}) + \nabla\cdot(w\ \text{kek1}) = k^{\text{KEK1}} s - k_d^{\text{KEK1}}\ \text{kek1} \end{cases}$$

1.3. Calibrating the egg chamber's growing dimensions and the nucleus movement

As we model the growing egg chamber, we have three moving parts to consider. The first is the dynamic position of the oocyte nucleus at the dorsal anterior corner of the oocyte as it grows. The second is the overall growth of the egg chamber, specifically the oocyte. The third is the posterior movement of the follicle cells as they become columnar around the oocyte nucleus and the stretched cells flatten around the nurse cells (Fig 1B). The respective time-dependent rates of movement, growth and shift, were extrapolated from measurements of the egg chamber at each stage (S1 Fig).

More specifically, measurements were made of the egg chamber's length and width (L_E and W_E), the oocyte length and width (L_O and W_O), and the length of the FCs (L_{FC}). The measurements were categorized into six different time points (S1 Fig), considering that each of the stages 7 to 10A lasts approximately 6 hours [4]. In addition, since egg chambers' morphologies change drastically at stages 8 and 9 within these six hours' time frame, we take measurements of S8 and S9 at two separate time points, early (here onward denoted by (S8E or S9E) and late (S8L or S9L), that are defined by the size of the egg chamber (S1 Fig). To summarize, we group the measurements of the egg chamber dimensions into six time-points: 3hr (corresponding to S7), 7.5hr (S8E), 10.5hr (S8L), 13.5hr (S9E), 16.5hr (S9L), 21hr (S10A). Values for all intermediate times were obtained by linear interpolation. S1 Fig A-B shows the measurements along the AP axis: L_O, L_{FC} and L_E. S1 Fig C-D shows the measurements along the DV axis: W_O and W_E.

For the growth of the egg chamber, the time-dependent big axis and small axis of the evolving prolate spheroid were set as: $L_{AP}(t) = \frac{1}{2} L_E(t)$ and $L_{DV}(t) = \frac{1}{2} W_E(t)$.

The dynamic position of the oocyte nucleus at each developmental stage was extrapolated from measurements taken from the anterior oocyte boundary to the posterior most point of the FCs (L_O).

Lastly, to account for the posterior movement of the follicle cells, measurements were taken from DAPI images for developmental stages 9, early and late, and 10A (L_{FC}), when follicle cell movement takes place.

1.4. Modeling the source of ligand

The level of detectable GRK secreted from the oocyte shows the output of GRK once it reaches the PVS. The size and the shape of the source of GRK, however, change throughout during the egg chamber development. We consider the shape of the ligand source as an input for the model, and thus calibrated this time and space-dependent source with experimental measurements (S2 Fig). At Stage 7, the oocyte nucleus encompasses the entire size of the oocyte; hence the size of the source is equivalent to the size of the egg chamber. As the oocyte grows, the size of the source changes and must be defined.

Measurements of the source were taken from inside of the oocyte from three different vantage points (S2 Fig A-B). At stages 9 early, 9 late, and 10A, direct measurements were taken from the anterior of the oocyte, the dorsal surface using confocal microscope cross-sections and a sagittal view (S2 Fig B). The shape of the source $\mathcal{s}(t)$ is then

modeled by the union of two triangles (in the variables (η,θ)), whose dimensions are calibrated from the experimental measurements, as follows:

$$\mathcal{s}(t) = \{(\eta,\theta) \mid \eta_0(t) \le \eta \le \eta_{\text{nucl}}(t) \text{ and } \theta \le \frac{\theta_0(t) - \theta_{\text{end}}(t)}{\eta_0(t) - \eta_{\text{end}}(t)}(\eta - \eta_{\text{nucl}}(t)) + \theta_{\text{end}}(t)\}$$

$$\cup \{(\eta,\theta) \mid \eta_{\text{nucl}}(t) \le \eta \text{ and } \theta \le \frac{\theta_{\text{nucl}}(t) - \theta_{\text{end}}(t)}{\eta_{\text{nucl}}(t) - \eta_{\text{end}}(t)}(\eta - \eta_{\text{end}}(t)) + \theta_{\text{end}}(t)\}.$$

The parameters $\theta_0(t)$, $\theta_{\text{nucl}}(t)$, $\theta_{\text{end}}(t)$, $\eta_0(t)$, $\eta_{\text{nucl}}(t)$ and $\eta_{\text{end}}(t)$ respectively represent the θ and η-coordinates of the signal at the oocyte anterior boundary (θ_0 and η_0), at the posterior of the oocyte nucleus (θ_{nucl} and η_{nucl}) and at the end of the gurken signal (θ_{end} and η_{end}). They are interpolated from experimental measurements of three ratios (Ratio (i), Ratio (ii) and Ratio (iii)) that characterize the source's shape at stages 9 early, 9 late, and 10A, presented in S2 Fig B.

More specifically, Ratio (i), which represents the ratio between the length of the signal and the length of the oocyte, allows us to fix the values of $\eta_{\text{end}}(t)$. Ratio (ii), which corresponds to the ratio between the width of the signal above the nucleus and the width of the oocyte at this same height, allows us to fix the values of $\theta_{\text{nucl}}(t)$. Ratio (iii), which corresponds to the ratio between the curved width of the signal just at the anterior of the nucleus and the half perimeter of the anterior-most part of the oocyte, allows to tune the parameters $\theta_0(t)$. The remaining parameters are built as follow: $\theta_{\text{end}}(t) = \theta_0(t)$ for $t \le 3$, $\theta_{\text{end}}(t) = 0$ for $t \ge 10.5$, and $\theta_{\text{end}}(t)$ is linearly interpolated between $\theta_0(t)$ and 0 for intermediate times. The parameter $\eta_0(t)$ is given by the η −coordinate of the anterior of the oocyte, which can be computed from the oocyte length $L_0(t)$ measured experimentally and presented in S1 Fig. The parameter $\eta_{\text{nucl}}(t)$ corresponds to the η − coordinate of the posterior of the oocyte nucleus, and is computed by adding the nucleus diameter to the z-coordinate of the anterior of the oocyte. Note that the values of the parameters $\theta_0(t)$, $\theta_{\text{nucl}}(t)$, $\theta_{\text{end}}(t)$, $\eta_0(t)$, $\eta_{\text{nucl}}(t)$ and $\eta_{\text{end}}(t)$ create a degenerate shape for earlier times ($t \le 10.5$), in which there is a single triangle.

The time-varying flux $V(t,\eta,\theta)$ is designed such that the total quantity of ligand at each time is proportional to the total surface of the egg chamber at that time (S2 Fig C). Denoting by σ_t the total surface area of the source at time t, by $\Sigma_t$the total surface of the egg chamber at time t and by V_t the flux of ligand at time t, we require: $\frac{V_t\,\sigma_t}{\Sigma_t} \equiv \frac{V_0\,\sigma_0}{\Sigma_0}$. We set:

$$\begin{cases} V(t,\eta,\theta) = V_t & \text{if the point } (\eta,\theta) \text{ belongs to the source } \mathcal{s}(t) \text{ at time } t \\ V(t,\eta,\theta) = 0 & \text{if the point } (\eta,\theta) \text{ is outside the source } \mathcal{s}(t) \text{ at time } t. \end{cases} \qquad (S6)$$

1.5. Parameterization of the perivitelline space

We represent the perivitelline space by a time-evolving prolate spheroid, parameterized at each time t by the coordinates (η,θ).

At time t, the perivitelline space is the set of points $\mathcal{S}(t) = \{P(t,\eta,\theta), \eta \in [0,\pi], \theta \in [0,2\pi]\}$ where $P(t,\eta,\theta)$ is given by:

$$P(t,\eta,\theta) = \begin{cases} x(t,\eta,\theta) = L_{DV}(t)\sin\eta\cos\theta \\ y(t,\eta,\theta) = L_{DV}(t)\sin\eta\sin\theta \\ z(t,\eta,\theta) = L_{AP}(t)\cos\eta \end{cases} \qquad (S7)$$

The parameters $L_{AP} = L_E/2$ and $L_{DV} = W_E/2$ denote the half lengths of the big and small axes, respectively, and correspond to the anterior-posterior and dorso-ventral half lengths of the egg chamber, which change dynamically with time. Their values are taken by interpolating the measurements done at each stage of oogenesis (S1 Fig). With these coordinates, the dorsal side of the egg chamber corresponds to $y = \theta = 0$ (S3 Fig A).

1.6. Calibrating the growth and shift vector fields

The knowledge of the egg chamber dimensions $L_{DV}(t)$ and $L_{AP}(t)$ at all time $t \in [0,24]$ allows us to determine a growth vector field v (symbolically represented by the blue arrows on S1 Fig E). Since many vector fields can be defined with these constraints, we design a simple vector field that preserves the prolate spheroid coordinates of a point when transporting it. This time-dependent vector field can be written as:

$$v(t,\eta,\theta) = \begin{pmatrix} L_{\mathrm{DV}}{}'(t)\sin\eta\cos\theta \\ L_{\mathrm{DV}}{}'(t)\sin\eta\sin\theta \\ L'_{\mathrm{AP}}(t)\cos\eta \end{pmatrix}.$$

With this choice, a point initially on the surface of the initial egg chamber $\mathcal{S}(0)$, parametrized by its prolate spheroidal coordinates (η_0, θ_0), has initial Euclidean coordinates $X(0) = (L_{\mathrm{DV}}(0)\sin\eta_0\cos\theta_0, L_{\mathrm{DV}}(0)\sin\eta_0\sin\theta_0, L_{\mathrm{AP}}(0)\cos\eta_0)$, and satisfies for all $t \geq 0$,

$$X(t) = (L_{\mathrm{DV}}(t)\sin\eta_0\cos\theta_0, L_{\mathrm{DV}}(t)\sin\eta_0\sin\theta_0, L_{\mathrm{AP}}(t)\cos\eta_0),$$

i.e. its prolate spheroidal coordinates (η_0, θ_0) are kept invariant.

Similarly, the shift vector field w is computed using the measurements of the length of the follicle cells $L_{\mathrm{FC}}(t)$. It is taken to be tangential to the prolate spheroid $\mathcal{S}(t)$, and to satisfy $w(t, \eta = 0, \theta) = 0$, so that the posterior-most point of the egg chamber (point (P) in S3 Fig A) is not shifted by the transformation. We also design w to be tangential to the prolate spheroid $\mathcal{S}(t)$, which gives the following expression:

$$w(t,\eta,\theta) = \alpha(t,\eta,\theta)\begin{pmatrix} L_{\mathrm{DV}}\cos\eta\cos\theta \\ L_{\mathrm{DV}}\cos\eta\sin\theta \\ -L_{\mathrm{AP}}\sin\eta \end{pmatrix}.$$

We determine the value of $\alpha(t)$ by assuming that the z-coordinate of a point on the prolate spheroid satisfies the following differential equation: $z'(t) = -\lambda(t)(z(t) - L_{\mathrm{AP}})$, ensuring that a point at the posterior pole of the prolate spheroid ($z(t) = L_{AP}$) satisfies $z'(t) = 0$. We further assume that the shift rate $\lambda(t)$ is piecewise constant, and satisfies $\lambda(t) = \lambda_i$ on each of the time intervals $[t_i, t_{i+1})$ given by the experimental measurements presented in Table 2.

Solving explicitly the differential equation for $z(t)$, we get $z(t) = L_{\mathrm{AP}} + e^{\lambda_i(t-t_i)}(z(t_i) - L_{\mathrm{AP}})$ for all $t \in [t_i, t_{i+1}]$. The knowledge of the length of the follicle cells $L_{\mathrm{FC}}(t_i)$ at the different time points (t_i) allows us to determine the rates (λ_i), and we further obtain the expression

$$w(t,\eta,\theta) = \lambda_i \frac{\cos\eta - 1}{\sin\eta} \begin{pmatrix} L_{\mathrm{DV}}(t)\cos\eta\cos\theta \\ L_{\mathrm{DV}}(t)\cos\eta\sin\theta \\ -L_{\mathrm{AP}}(t)\sin\eta \end{pmatrix} \text{ for all } t \in [t_i, t_{i+1}).$$

Note that many other tangential vector fields could be designed by modifying some of the assumptions made above. The advantage of this specific approach is that the experimental measurements $L_{\mathrm{FC}}(t_i)$ can be fitted directly into the equation for the z-coordinate, thus limiting the number of operations. In the absence of more accurate experimental data, this simple approach is sufficient to capture the overall dynamics of the follicle cells' shift, but it could easily be adapted to fit new data in the future.

Section S2: Numerical methods

2.1. Numerical discretization of the domain

A- Initial Domain:

The spheroidal parameterization is not a diffeomorphism from $[0,\pi] \times [0,2\pi]$ to $\mathcal{S}(t)$. Indeed, for all $\theta \in [0,2\pi]$, $(x,y,z)(t,0,\theta) = (0,0,L_{AP}(t))$ and $(x,y,z)(t,\pi,\theta) = (0,0,-L_{AP}(t))$.

Because of this, the spheroidal mesh built from these coordinates contains two singularities, or overlapping points, at $(0,0,L_{AP})$ and $(0,0,-L_{AP})$. As a consequence, the discretization points close to the poles $z = L_{AP}$ and $z = -L_{AP}$ are much closer than those towards the equator $z = 0$. This characteristic implies that the spheroidal mesh has irregular cell sizes, which makes it ill-suited for the finite-differences approximation of the diffusion operator. For this reason, we present another system of coordinates that provides a more regular discretization of the domain.

A way to construct a more regular discretization of the spheroid is to divide it into several subdomains, each endowed with their own coordinate system. We developed the cubed spheroid coordinate system by extending the "cubed sphere" approach described in [Nair et al] in the context of the discontinuous Galerkin numerical scheme (S3 Fig B-E).

In order to extend the cubed sphere parameterization, we define a homeomorphism between each point of the prolate spheroid P of small axis length L_{DV} and big axis length L_{AP} and the sphere S_{AP} of radius L_{AP} given by the equation $x^2 + y^2 + z^2 = L_{AP}^2$.

Let Φ_S be defined by $\Phi_S: S_{AP} \to P$, with

$$\Phi_S: (x_s, y_s, z_s) \mapsto (x,y,z) = \left(\frac{L_{DV}}{L_{AP}} x_s, \frac{L_{DV}}{L_{AP}} y_s, z_s\right) \qquad (S8)$$

The homeomorphism Φ_S^{-1} transforms each point P of the prolate spheroid P to a point P_s of the sphere S_{AP} by projection along the direction $\overrightarrow{P_z P}$, where P_z has coordinates $(0,0,z)$.

The division of the sphere into "cubed" subdomains was introduced in [Nair et al]. We recall it here.

Let C_a be the cube of side $2a$ inscribed in S_{AP}, oriented such that the 3D Cartesian

axes are orthogonal to its faces (see S3 Fig C). By definition, $a = \frac{1}{\sqrt{3}} L_{AP}$. We define the mapping $\Phi_C: P_s \in S_{AP} \mapsto P_s \in C_a$ by projection along the direction $\overrightarrow{OP_S}$. Then the point P_s can be parametrized by some coordinates (x_C, y_C) and the parameterization depends on the face of C_a. Let F_0 be the face of C_a belonging to the plane $z = a$.

Let $(x_C, y_C) \in [-a, a] \times [-a, a]$ be the local coordinates on F_0 (see S3 Fig C). Geometrically, we have the following relation between (x_C, y_C) and (x_s, y_s, z_s):

$$\begin{cases} x_C = \dfrac{a\, x_s}{z_s} \\ y_C = \dfrac{a\, y_s}{z_s}. \end{cases} \qquad (S9)$$

Then the mapping ${\Phi_C}^0: F_0 \to S_{AP}$ mapping the cube's side F_0 to the prolate spheroid is defines by

$$\begin{aligned} {\Phi_C}^0: (x_C, y_C) &\mapsto (x_s, y_s, z_s) \\ &= \left(\frac{x_C L_{AP}}{\sqrt{a^2 + x_C^2 + y_C^2}}, \frac{y_C L_{AP}}{\sqrt{a^2 + x_C^2 + y_C^2}} \frac{a L_{AP}}{\sqrt{a^2 + x_C^2 + y_C^2}} \right) \end{aligned} \quad (S10)$$

Similar parametrizations can be given by defining local coordinate systems on the other faces of the cube. Composing ${\Phi_C}^0$ and Φ_S gives a parameterization of the prolate spheroid that we name *cubed spheroid parameterization*. The respective images of the faces F_i of C_a by $\Phi_S \circ {\Phi_C}^i$ (for $i \in \{0, \dots, 5\}$) divide the spheroid into 6 domains F_i . For instance, the local coordinates on F_i define a subdomain of S that we denote by D_0 and that can be parameterized combining the two maps as follows:

$$\begin{aligned} (x, y, z) &= \Phi_S \circ {\Phi_C}^0 (x_C, y_C) \\ &= \left(\frac{x_C L_{DV}}{\sqrt{a^2 + x_C^2 + y_C^2}}, \frac{y_C L_{DV}}{\sqrt{a^2 + x_C^2 + y_C^2}}, \frac{a L_{AP}}{\sqrt{a^2 + x_C^2 + y_C^2}} \right) \end{aligned} \quad (S11).$$

In practice, we restrict ourselves to a quarter prolate spheroid (S3 Fig B-E). In this case, only four faces of the cube need to parameterize it, which divides the spheroid into four subdomains, denoted by D_0, D_1, D_2 and D_3 (S3 Fig F-G). The coordinates on the total prolate spheroid can be obtained by symmetry. We create a cubed spheroidal mesh by discretizing these coordinates.

B- Time evolution of the domain:

Once the initial domain is discretized, it is transported by the flow of a growth vector field v. In our approximation, the growth vector field transforms the initial prolate spheroid into a new prolate spheroid. We choose to discretize the prolate spheroid at time t by the push-forward of the initial mesh. More specifically, given the initial mesh characterized by a set of points $(X_i^0, Y_i^0, Z_i^0)_{i=1,\dots,N}$, the mesh at time t will be given by the same number of points, whose coordinates satisfy (in a first-order approximation):

$$\begin{pmatrix} X_i^{t+\Delta t} \\ Y_i^{t+\Delta t} \\ Z_i^{t+\Delta t} \end{pmatrix} = \begin{pmatrix} X_i^t \\ Y_i^t \\ Z_i^t \end{pmatrix} + \Delta t \, v \left(t, \begin{pmatrix} X_i^t \\ Y_i^t \\ Z_i^t \end{pmatrix} \right).$$

We stress the fact that this method is feasible due to two main specificities of our problem.

1) Although the growth of the egg chamber modifies its geometry, it remains possible to approximate its shape by a prolate spheroid at all time (characterized by the time-evolving lengths $(L_{AP}(t), L_{DV}(t))$, hence it can be discretized by the cubed-sphere mesh. This property would also hold for most smooth surfaces homeomorphic to the sphere that do not evolve to become singular.
2) During the time-span of interest (S7 to S10A of oogenesis), the dimensions of the egg chamber increase by a factor of 3 to 4. This implies that each surface element's dimensions increase by a factor of 3 to 4, and thus the discretization of the prolate spheroid by push-forward of the initial mesh is coarser for S10A than for S7. This can be compensated by taking a finer mesh at S7, in order to ensure a good discretization at S10A. However, if the growth were to change drastically the order of magnitude of the dimensions of the domain, another discretization method would have to be considered, such as an adaptive mesh.

2.2. Numerical implementation by operator splitting.

We solved the system of coupled PDEs (5)-(6)-(7) by operator splitting, using the forward Euler method. Each time step is further divided into four sub-steps in which we treat independently:

- diffusion: the time-varying Laplace-Beltrami operator is approximated with finite differences in each zone;
- growth: all variables are pushed-forward via the vector field v to the new prolate spheroid;
- shift of follicle cells: all variables except the ligand L are pushed-forward towards the oocyte posterior via the vector field w;
- reactions: the system is now reduced to coupled ODEs

We present a more detailed explanation of the numerical treatment of the diffusion and divergence terms below.

2.3. Numerical approximation of the Laplace-Beltrami operator

Given local coordinates $(x_i)_{i\in\{1,\dots,d\}}$ and a metric tensor $(g_{ij})_{(i,j)\in\{1,\dots,d\}^2}$, the Laplace-Beltrami operator is given by the explicit expression:

$$\Delta_{LB} f = \frac{1}{\sqrt{|g|}} \sum_{i=1}^{d} \partial_{x_i} \left(\sqrt{|g|}\, g^{ij}\, \partial_{x_j} f \right) \qquad (S12)$$

Where the tensor $(g^{ij})_{(i,j)\in\{1,\dots,d\}^2}$ denotes the inverse of $(g_{ij})_{(i,j)\in\{1,\dots,d\}^2}$, and $|g|$ denotes its determinant.

Using the cubed spheroidal parameterization, one can compute the metric tensor induced from the Riemannian metric in each subdomain.

Let $r = (x, y, z)$. Then in D_0, the metric tensor G_0 is given by:

$$G_0 = \begin{pmatrix} g_{11}^0 & g_{12}^0 \\ g_{12}^0 & g_{22}^0 \end{pmatrix} \qquad (S13)$$

where

$$\begin{cases} g^0_{11} = |\partial_{x_C} r|^2 = \dfrac{1}{(a^2 + x_C^2 + y_C^2)^3}\left(L_{DV}^2 (a^2 + y_C^2)^2 + L_{DV}^2 x_C^2 y_C^2 + L_{AP}^2 a^2 x_C^2\right) \\ g^0_{22} = |\partial_{y_C} r|^2 = \dfrac{1}{(a^2 + x_C^2 + y_C^2)^3}\left(L_{DV}^2 (a^2 + x_C^2)^2 + L_{DV}^2 x_C^2 y_C^2 + L_{AP}^2 a^2 y_C^2\right) \quad (S14) \\ g^0_{12} = \langle \partial_{x_C} r, \partial_{y_C} r \rangle = \dfrac{1}{(a^2 + x_C^2 + y_C^2)^3}\left(-L_{DV}^2 (2a^2 + x_C^2 + y_C^2)^2 + L_{AP}^2 a^2 x_C y_C\right). \end{cases}$$

The expression of the metric tensor allows to compute the Laplace-Beltrami operator in the cubed spheroid coordinates in each domain.

Using cubed spheroidal coordinates requires subdividing the prolate spheroid into several subdomains, and treating the interfaces between domains with appropriate boundary conditions. Notice that due to the symmetry of the source function with respect to the (X0Z) plane, the full system is symmetric with respect to the (X0Z) plane. It is then sufficient to solve numerically the system of equations on a half prolate spheroid, and to recover the solution on the full domain by symmetry with respect to (X0Z). The boundaries between domains are treated with Dirichlet boundary conditions. The boundaries of the quarter spheroid inscribed in the (X0Z) plane are treated with Neumann boundary conditions, for reasons of symmetry (see S3 Fig G).

2.4. Numerical treatment of the divergence terms

We bring our attention to the treatment of the divergence operators present in Equations (5) and (7) (also (S1) and (S2)).

To solve a continuity equation of the type $\partial_t f(t,x) = \nabla \cdot \big(V(t,x) f(t,x)\big)$, we adopt a Lagrangian approach. A moving surface element S_t belonging to the prolate spheroid $\mathcal{S}(t)$ at time t is transported to a new surface element S_{t+dt} belonging to the prolate spheroid $\mathcal{S}(t+dt)$ at time $t+dt$. By mass conservation, it holds

$$\frac{d}{dt}\int_{S_t} f(t,x)\, dS = 0,$$

and for small time, respectively denoting by c_t and c_{t+dt} the centers of mass and by $|S_t|$ and $|S_{t+dt}|$ the measures of S_t and S_{t+dt}, it holds:

$$f(t,c_t)\,|S_t| = f(t,c_{t+dt})\,|S_{t+dt}|,$$

which we use to compute the value of $f(t,c_{t+dt})$ from the knowledge of $|S_t|$, $|S_{t+dt}|$ and $f(t,c_t)$.

Since the domain (a prolate spheroid) is discretized using the Cubed Sphere mesh, each surface element can be approximated by a parallelogram in $\mathbb{R}^3$, and its area is computed in a classical way from the knowledge of the coordinates of its four vertices, which correspond to the mesh points.

Importantly, the two vector fields v and w have two different roles. The growth vector field v acts on the geometry of the domain, while the shift vector field w transports the variables tangentially. For this reason, we treat them in two different ways.

A- Growth vector field

The treatment of the growth vector field is divided into two steps.

Step 1: The Cubed Sphere mesh is pushed-forward by the growth vector field v (see Section **Numerical Discretization,** Time evolution of the domain). Given a point (X_i^t, Y_i^t, Z_i^t) of the initial mesh, its push-forward by the flow of v (in a first-order approximation) is given by:

$$\begin{pmatrix} X_i^{t+\Delta t} \\ Y_i^{t+\Delta t} \\ Z_i^{t+\Delta t} \end{pmatrix} = \begin{pmatrix} X_i^t \\ Y_i^t \\ Z_i^t \end{pmatrix} + \Delta t\, v\left(t, \begin{pmatrix} X_i^t \\ Y_i^t \\ Z_i^t \end{pmatrix} \right).$$

Step 2: The areas of the old and new surface elements are computed and used to compute the new value of the density f after growth.

$$f\left(t+\Delta t, \begin{pmatrix} X_i^{t+\Delta t} \\ Y_i^{t+\Delta t} \\ Z_i^{t+\Delta t} \end{pmatrix} \right) = \frac{|S_t^i|}{|S_{t+dt}^i|}\, f\left(t, \begin{pmatrix} X_i^t \\ Y_i^t \\ Z_i^t \end{pmatrix} \right)$$

B- Shift vector field

The shift vector field does not modify the mesh of the Prolate Spheroid, and transports the densities tangentially. We divide the computation of the push-forward of a density f in three steps:

Step 1: An auxiliary mesh is computed by the push-forward of the tangential vector field:

$$\begin{pmatrix} \tilde{X}_i^t \\ \tilde{Y}_i^t \\ \tilde{Z}_i^t \end{pmatrix} = \begin{pmatrix} X_i^t \\ Y_i^t \\ Z_i^t \end{pmatrix} + \Delta t\, w\left(t, \begin{pmatrix} X_i^t \\ Y_i^t \\ Z_i^t \end{pmatrix} \right).$$

Step 2: The areas of the original and auxiliary surface elements are used to compute the value of the auxiliary density f evaluated at the vertices of the auxiliary mesh:

$$\tilde{f}\left(t+\Delta t, \begin{pmatrix} \tilde{X}_i^t \\ \tilde{Y}_i^t \\ \tilde{Z}_i^t \end{pmatrix} \right) = \frac{|S_t^i|}{|\tilde{S}_t^i|}\, f\left(t, \begin{pmatrix} X_i^t \\ Y_i^t \\ Z_i^t \end{pmatrix} \right).$$

Step 3: The values of the density at the original mesh vertices are computed by interpolating the auxiliary density.

Section S3: Extrapolation of parameter values from intensity measurements:

Since the intensity values cannot be measured directly, the inhibitors' strengths γ_{Kek} and γ_{Sty} were determined by comparing simulation results for different values of these two parameters by measurements of dpERK immunostainings as an output of the signaling intensity. Intensity measurements for dpERK were done at early and late Stages 9 and Stage 10A along the anterior-posterior axis and the dorso-ventral axis for wild-type, 1px and 4px flies (px represents the copy number of *grk* in the genome, i.e. the wild type has two copies, hence 2px).

Simulations were run with our model for wild-type (2px), 1px and 4px, for six different values of γ_{Kek} and seven different values of γ_{Sty}, ranging two orders of magnitude. This provided us with 42 AP and 42 DV intensity plots for each of the three stages and each of the three perturbations. These data allowed a rich information base to select the combination of parameters γ_{Kek} and γ_{Sty} that provide the closest results to the experimental measurements.

This was done by computing a cost function for each of the 42 combinations of parameters. Let us denote by ${u_{AP}}^i, {v_{AP}}^i, {w_{AP}}^i, {u_{DV}}^i, {v_{DV}}^i$, and ${w_{DV}}^i$ respectively the 6 AP and DV curves at S10A, corresponding to wild-type, 1px and 4px, for the i-th combination of parameters, after normalization by the constant K_i. The corresponding experimental profiles are respectively denoted by ${U_{AP}}^i, {V_{AP}}^i, {W_{AP}}^i, {U_{DV}}^i, {V_{DV}}^i$, and ${W_{DV}}^i$. Then the cost Γ^{i} of the i-th combination of parameters is:

$$\Gamma^{\text{i}} = \left\|{u_{AP}}^i - {U_{AP}}^i\right\|_{L^1} + \left\|{v_{AP}}^i - {V_{AP}}^i\right\|_{L^1} + \left\|{w_{AP}}^i - {W_{AP}}^i\right\|_{L^1} + \left\|{u_{DV}}^i - {U_{DV}}^i\right\|_{L^1} + \left\|{v_{DV}}^i - {V_{DV}}^i\right\|_{L^1} + \left\|{w_{DV}}^i - {W_{DV}}^i\right\|_{L^1},$$

where $\|\cdot\|_{L^1}$ denotes the L^1 norm in 1D:

$$\left\|{u_{AP}}^i - {U_{AP}}^i\right\|_{L^1} := \int_0^{100} \left|{u_{AP}}^i(\xi) - {U_{AP}}^i(\xi)\right| d\xi.$$

Notice that both experimental and simulation curves are horizontally normalized so that $\xi \in [0,100]$ represents the percentage of the length of the measured experimental data.

The parameters $\gamma_{\text{Sty}} = 500 \times 10^2$ and $\gamma_{\text{kek}} = 10 \times 10^2$ showed the best fit to the experimental measurements.

Bibliography

[1] M. Pribyl, C. B. Muratov, S. Y. Shvartsman, Discrete models of autocrine cell communication in epithelial layers. *Biophysical journal* **84**, 3624-3635 (2003).

[2] M. Pribyl, C. B. Muratov, S. Y. Shvartsman, Long-range signal transmission in autocrine relays. *Biophysical journal* **84**, 883-896 (2003).

[3] S. Sigismund *et al.*, Clathrin-mediated internalization is essential for sustained EGFR signaling but dispensable for degradation. *Dev Cell* **15**, 209-219 (2008).

[4] A. C. Spradling, *Developmental genetics of oogenesis. In: The Development of Drosophila melanogaster* (Plainview: Cold Spring Harbor Laboratory Press., 1993), pp. 1-70.

[5] L. A. Goentoro *et al.*, Quantifying the Gurken morphogen gradient in Drosophila oogenesis. *Dev Cell* **11**, 263-272 (2006).
