# Supplementary figures and images for: Correction: A computational framework to study EGFR signaling distribution in egg chambers during dynamic interactions between soma and germline
Source: PLoS Comput Biol. 2026 Apr 3;22(4):e1014155. doi: 10.1371/journal.pcbi.1014155 (PMC13048367; doi:10.1371/journal.pcbi.1014155)

**Figure S1**

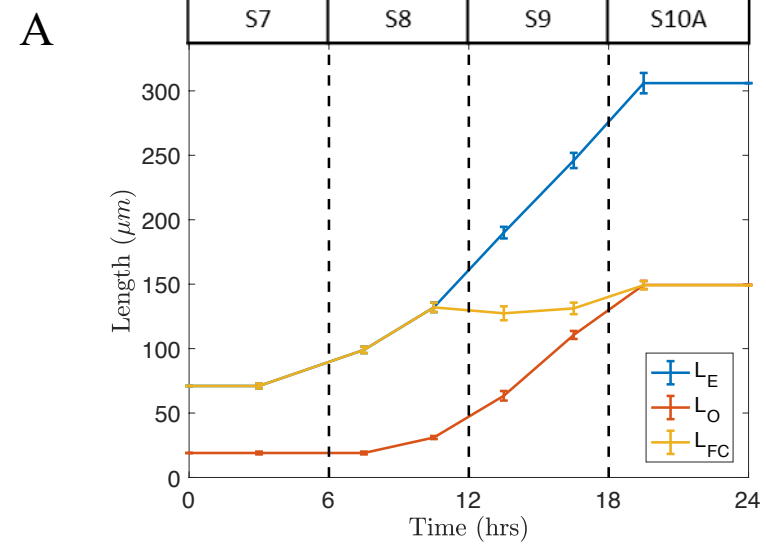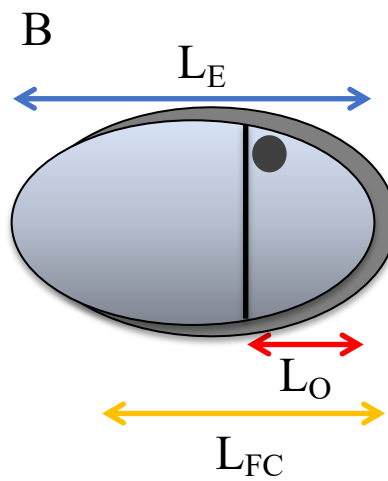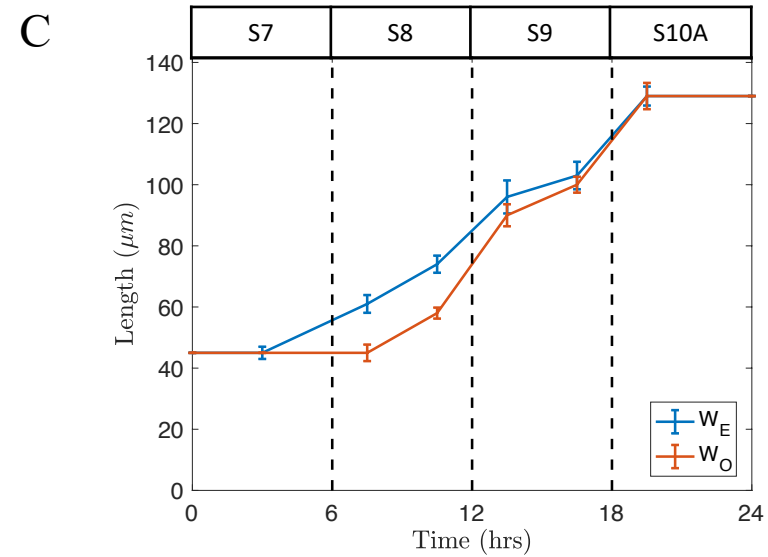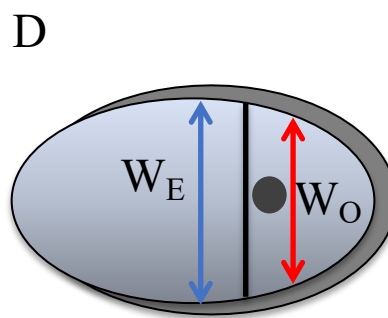

Figure S1

E

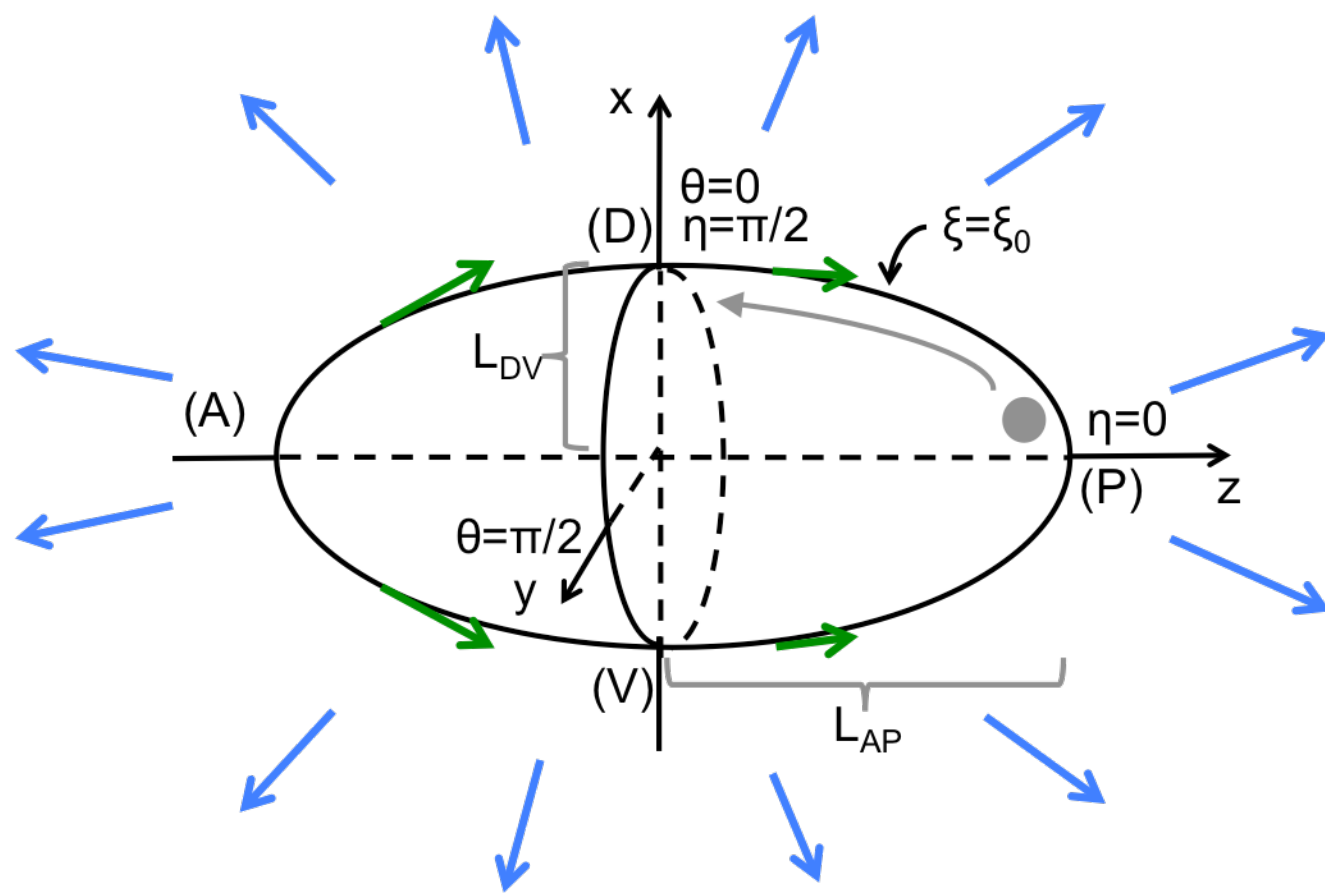

Supplement: S1 Fig — Data was collected at 6 consecutive time-points: 3hr (S7), 7.5hr (S8E), 10.5hr (S8L), 13.5hr (S9E), 16.5hr (S9L), 19.5hr (S10AE). A. Three measurements were taken in the AP direction: the total length of the egg chamber LE, the length of the oocyte L0, and the length of the follicle cells LFC. B. A cartoon schematic showing positions of measurements along the AP axis. C. Two measurements were taken in the DV direction: the egg chamber width WE and the oocyte width near the oocyte nucleus W0. D. A cartoon schematic showing positions of measurements along the DV axis. Note that the semi-axes of the prolate spheroid modeling the oocyte then correspond to half of the egg chamber length and width: and E. A cartoon schematic showing the different morphological transformations taken into account. The growth vector field, perpendicular to the egg chamber surface, is schematized by blue arrows. The follicle cells’ shift, tangent to the egg chamber surface, is represented by green arrows. The oocyte nucleus’ movement from (P) to (D), in the plane (xOz) is indicated by a grey arrow. (PDF) [file pcbi.1014155.s001.pdf]

Figure S2

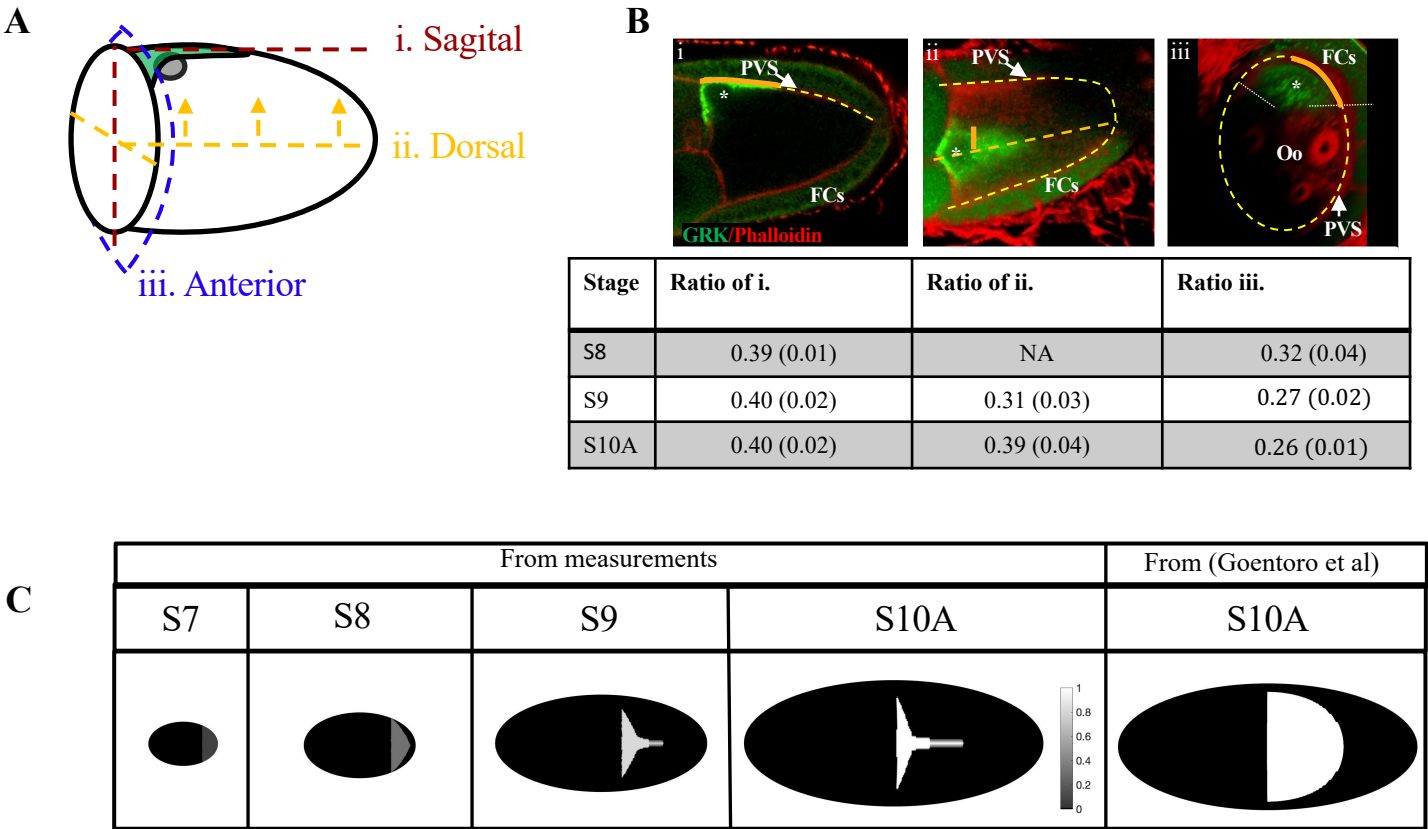

Supplement: S2 Fig — A. A cartoon depicting the vantage points from where source measurements were taken at the (i) Sagital, denoted by red dotted line, (ii) Dorsal, denoted by yellow dotted line, and (iii) Anterior, denoted by blue dotted line. B. Immunohistochemistry stainings from vantage points of the egg chamber at S10A: (i) Sagital (ii) Dorsal measurement from a ventral view (iii) Anterior boundary of oocyte. Corresponding table provides ratios of length of source compared to total length of domain (i.e. Sagital (Sag), Dorsal (D), Anterior (A)) at S8 (n=6 D,A), S9 (n=8 for D, Sag,; n =10 A), and S10A (n=5 for D,Sag, n=9 for A). More precisely, Ratio (i) corresponds to the ratio of the dorsal length of the source with respect to the total (curved) length of the oocyte. Ratio (ii) corresponds to the ratio of the width of the source at the posterior of the nucleus with respect to the width of the oocyte at the posterior of the nucleus. Ratio (iii) corresponds to the ratio between the curved width of the signal at the anterior of the nucleus and the half perimeter of the oocyte at the anterior of the nucleus. C. Representation of the numerical implementation of the source and table of experimental measurements of the source at specific stages. At S10A we show the current numerical implementation of the source compared to the source used in (Goentoro et al., 2006). (PDF) [file pcbi.1014155.s002.pdf]

Figure S3

A

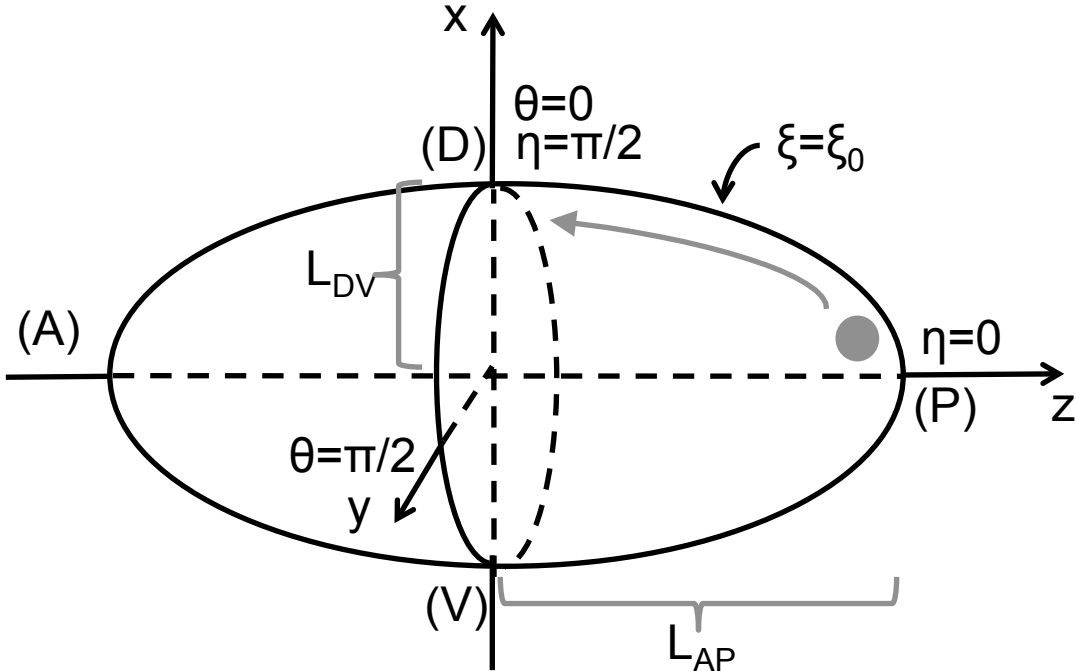

Figure S3

B

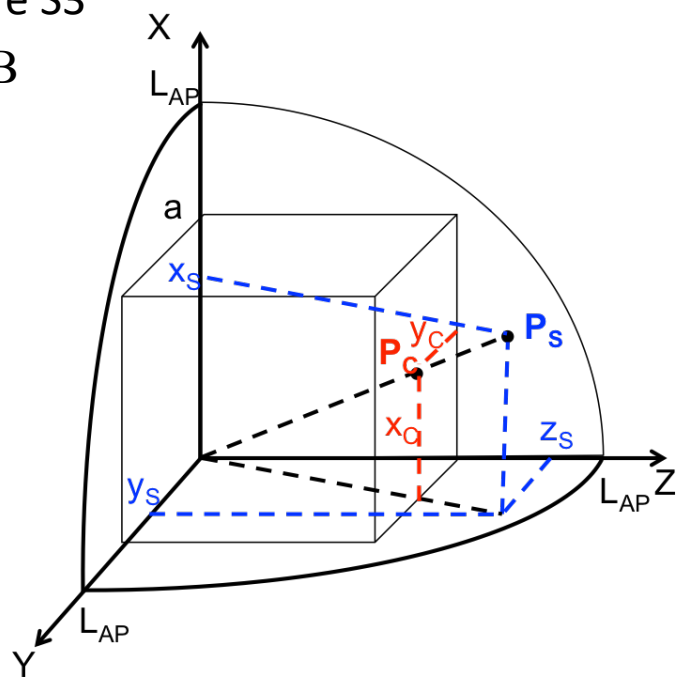

C

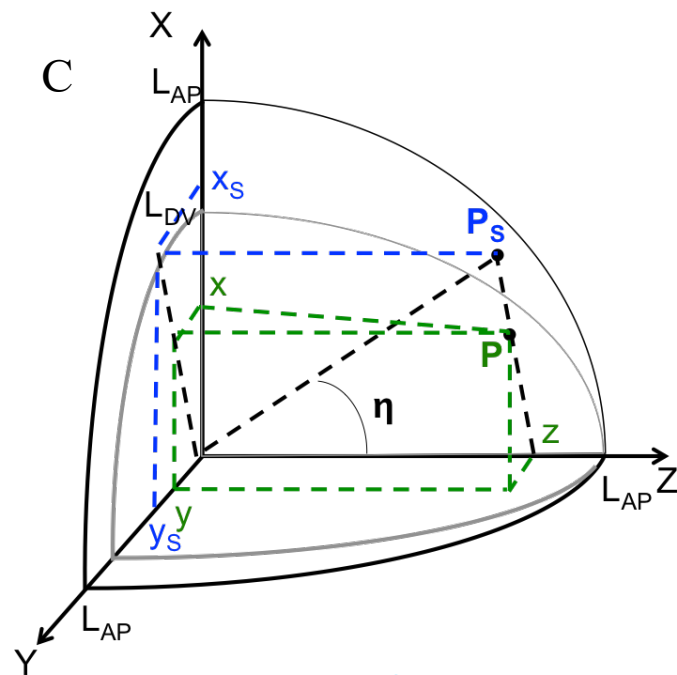

D

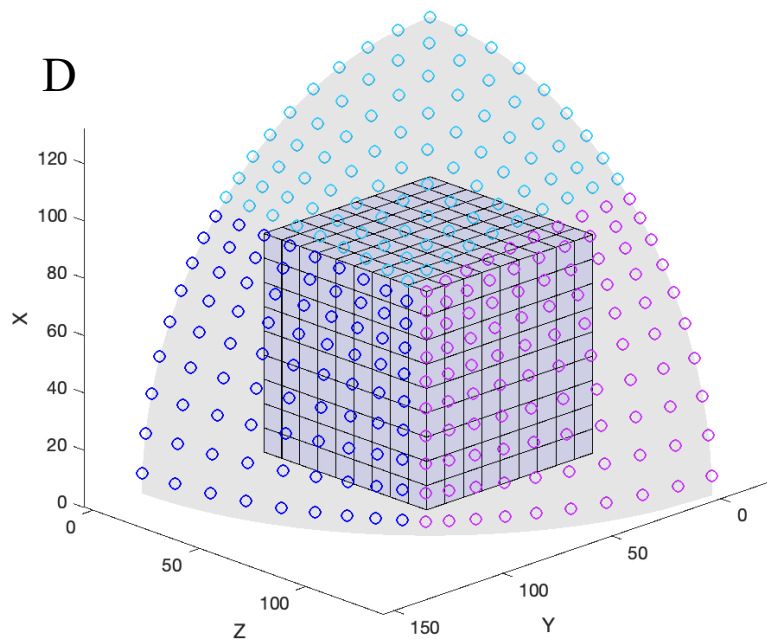

E

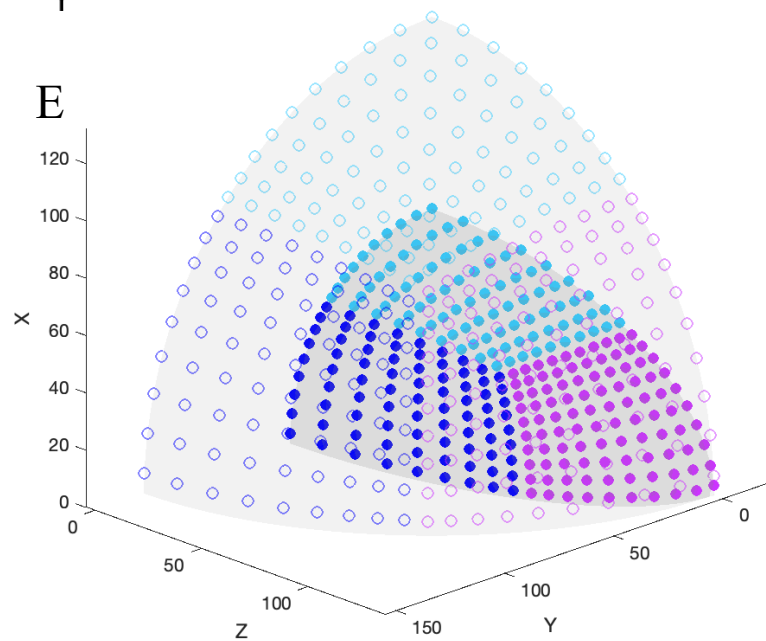

Figure S3

F

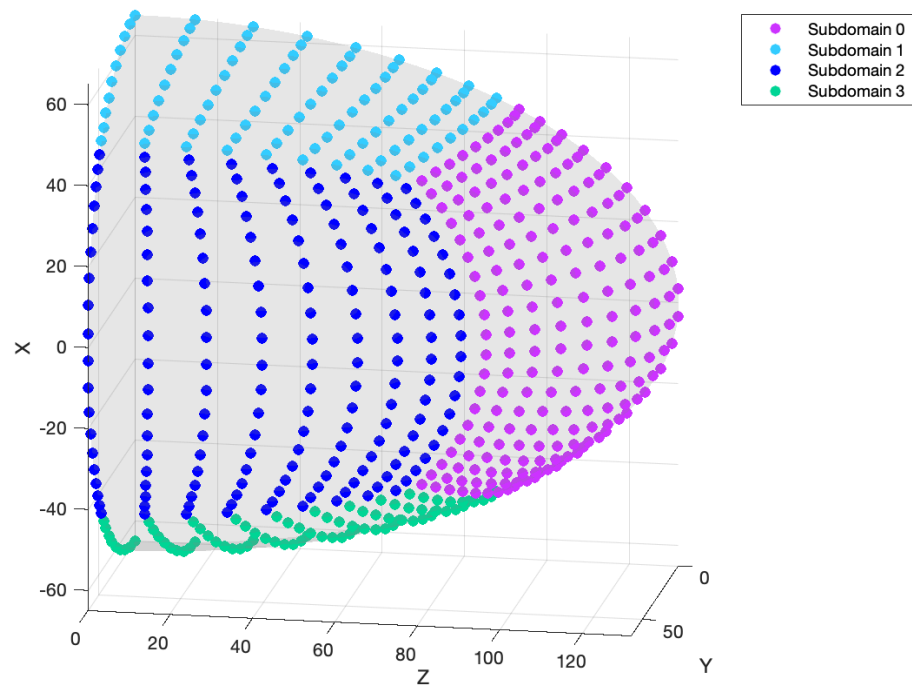

G

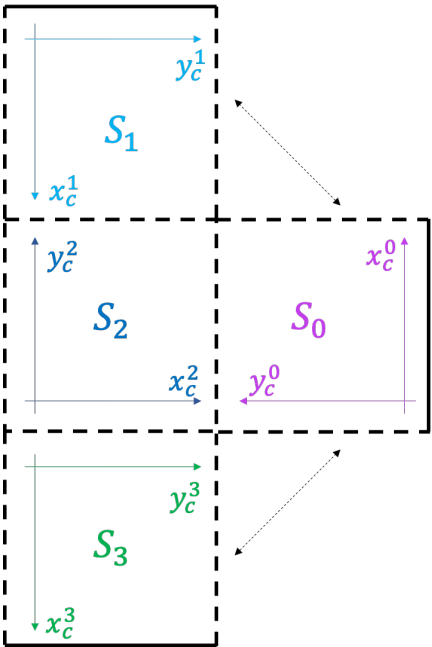

Supplement: S3 Fig — Parametrization of the prolate spheroid representing the egg-chamber at a given time t by (𝜂, 𝜃) ∈ [0, 𝜋] × [0,2𝜋]. The lengths of its semi-axes are 𝐿𝐴𝑃(𝑡) (along the z- axis), and 𝐿𝐷𝑉(𝑡) (along the x and y-axes). The posterior (𝑃) corresponds to 𝜂 = 0, and the anterior (𝐴) to 𝜂 = 𝜋. The dorsal side (𝐷) corresponds to 𝜃 = 0 and the ventral side (𝑉) to 𝜃 = 𝜋. The oocyte nucleus, represented by the gray circle, migrates from the posterior to the dorsal anterior of the spheroid. B-E. Construction of the cubed spheroidal mesh. The cubed spheroidal mesh is obtained by a two-step transformation, from the cube 𝐶𝑎 to the sphere 𝑆𝐴𝑃, and from the sphere 𝑆𝐴𝑃 to the spheroid 𝑃. Consider the sphere of radius 𝑆𝐴𝑃 centered at 0 and let 𝐶𝑎 be its inscribed cube of side, also centered at 0. Each side of 𝐶𝑎 is discretized by a regular orthonormal mesh (D). Then the cubed spherical mesh of 𝑆𝐴𝑃 is obtained by taking the radial projection of the mesh of 𝐶𝑎 onto 𝑆𝐴𝑃 (D and B): each vertex 𝑃C(𝑥𝑐, 𝑦𝑐, 𝑧𝑐) ∈ 𝐶𝑎 of the mesh is projected radially onto 𝑆𝐴𝑃, giving the point 𝑃𝑆(𝑥𝑆, 𝑦𝑆, 𝑧𝑆) ∈ 𝑆𝐴𝑃. By this transformation, the sphere 𝑆𝐴𝑃 is meshed by the cubed sphere projection. Secondly, each vertex 𝑃𝑆 ∈ 𝑆𝐴𝑃 of the cubed spherical mesh is projected onto the prolate spheroid 𝑃, orthogonally to the 𝑧-axis (C). This transformation defines the cubed spheroidal mesh (E). F-G. Division of the prolate spheroid 𝑃 into subdomains. From the cubed spheroid mesh construction, each quarter spheroid is divided into 4 subdomains, corresponding to four sides of the cube. In S3 Fig F, we represent the quarter spheroid contained in the region {(x, y, z) ∈ ℝ3, 𝑦 ≥ 0, 𝑧 ≥ 0}. It is divided into the subdomains 𝑆0 (covering the posterior pole), 𝑆1 (dorsal region), 𝑆2 (lateral region) and 𝑆3 (ventral region). S3 Fig G shows the boundary conditions implemented at the subdomain interfaces. Dotted lines represent matching boundary conditions, whereas continuous lines indicate Neumann boundary conditions (due to the symme [file pcbi.1014155.s003.pdf]
