# Supplementary material for: Correction: A computational framework to study EGFR signaling distribution in egg chambers during dynamic interactions between soma and germline
Source: PLoS Comput Biol. 2026 Apr 3;22(4):e1014155. doi: 10.1371/journal.pcbi.1014155 (PMC13048367; doi:10.1371/journal.pcbi.1014155)
Supplement: S4 Fig — A. Intensity profiles of GRK for varying GRK copy numbers at 2x (OreR), 4x, and 1x along the AP (top row) and DV (bottom row). B. Intensity profiles of dpERK for varying GRK copy numbers at 2x (OreR), 4x, and 1x along the AP (top row) and DV (bottom row). C. Intensity profiles of GRK for three genetic backgrounds: wild-type (OreR), STY- RNAi, and EGFR-RNAi along the AP (top row) and DV (bottom row). D. Intensity profiles of dpERK for three genetic backgrounds: wild-type (OreR), STY- RNAi, and EGFR-RNAi along the AP (top row) and DV (bottom row). (PDF) [file pcbi.1014155.s004.pdf]

Figure S4.

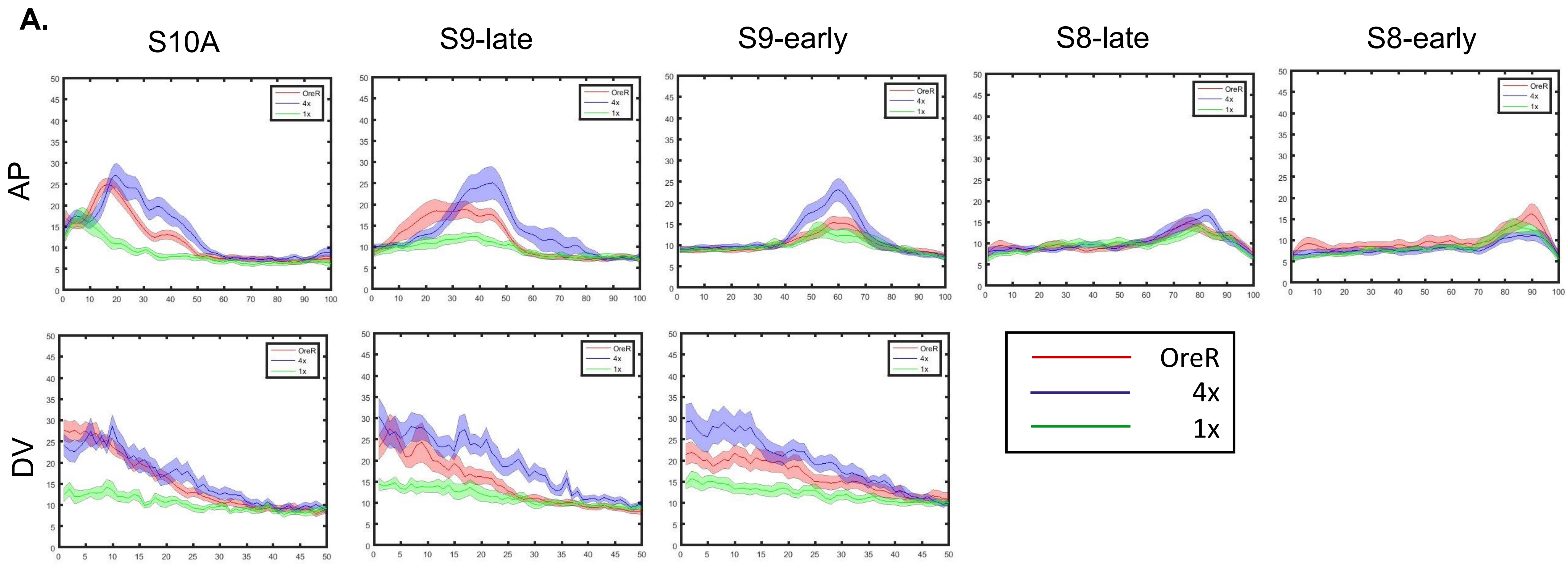

Figure S4.

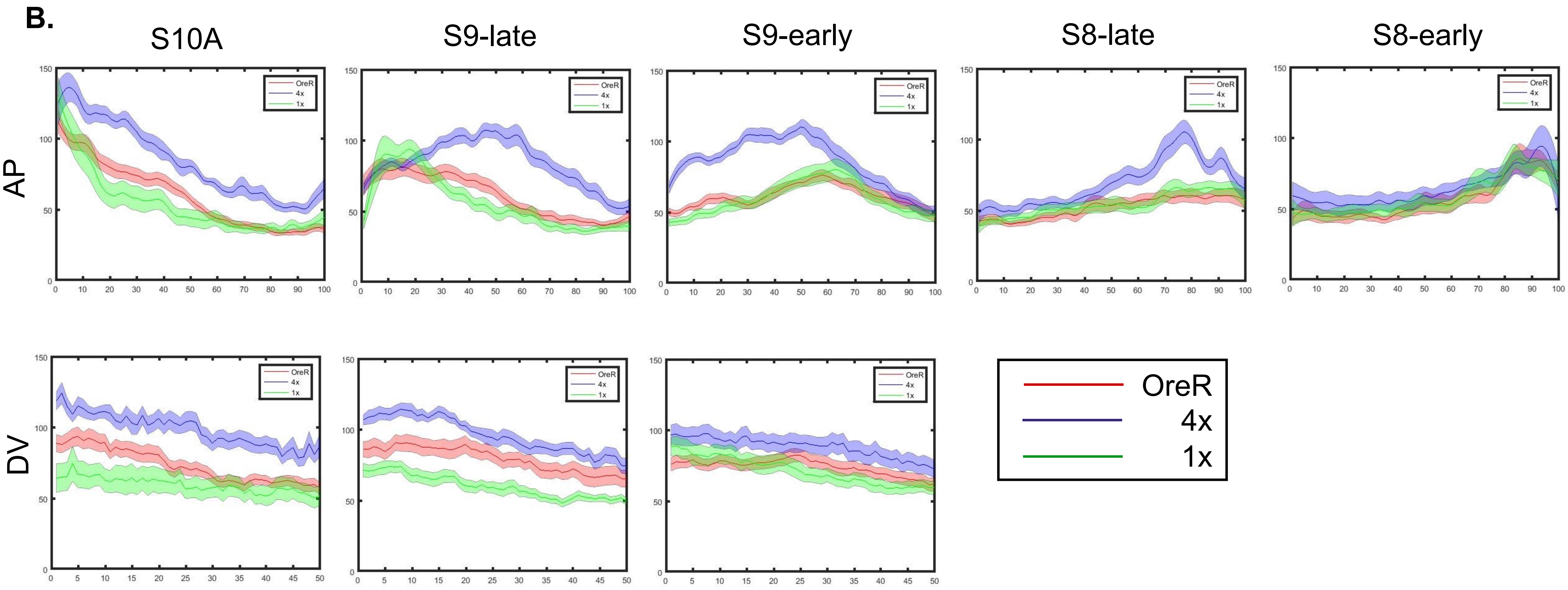

Figure S4.

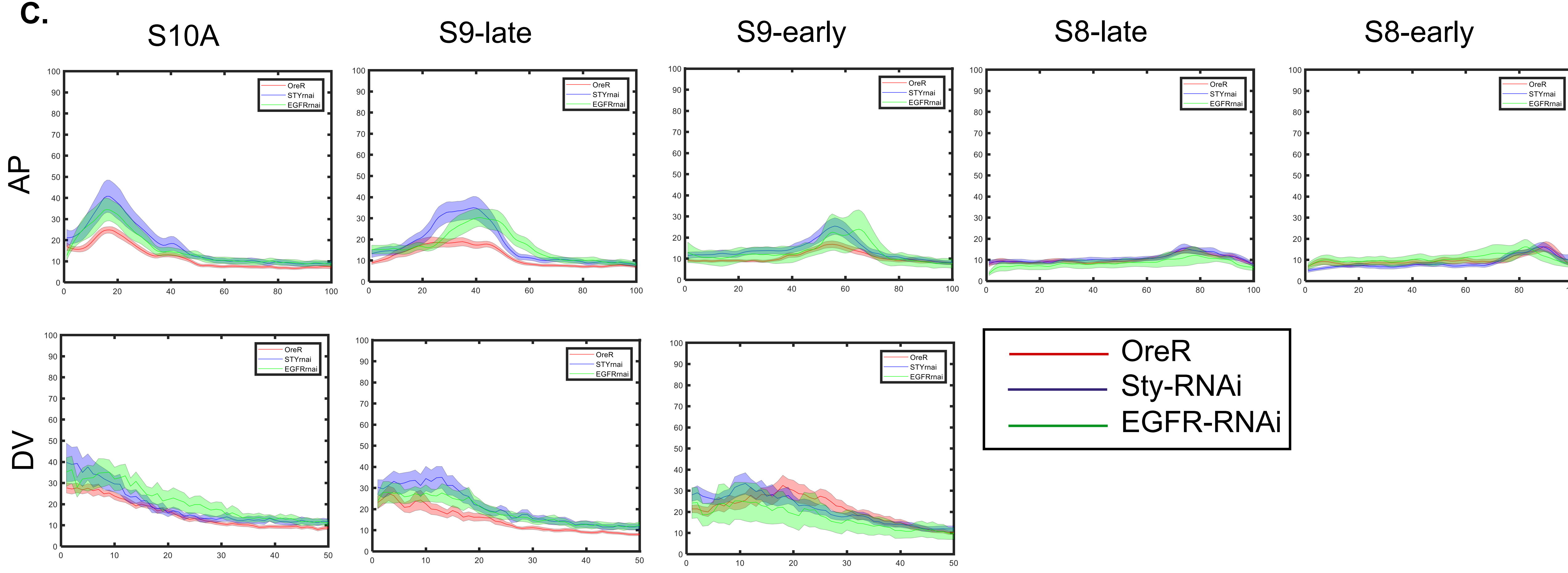

C.
S10A
S9-late
S9-early
S8-late
S8-early
AP
DV
OreR
STYrnai
EGFRrnai
OreR
Sty-RNAi
EGFR-RNAi

Figure S4.

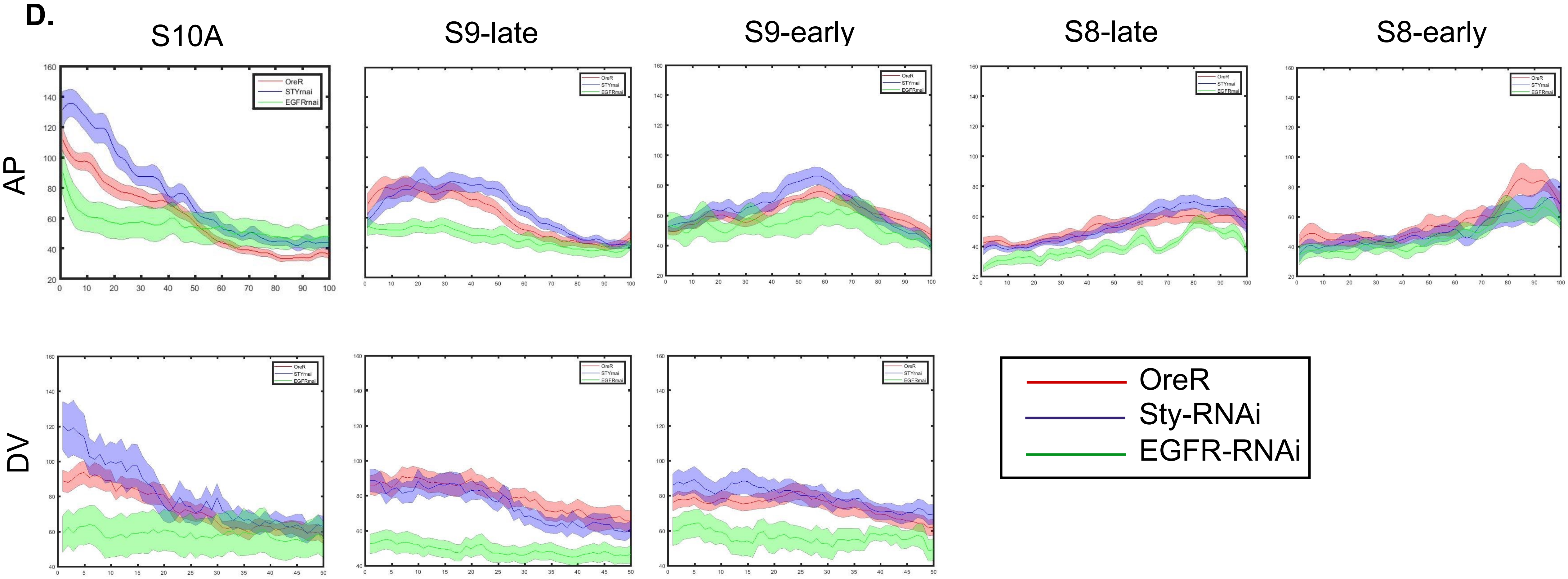
D.
S10A
S9-late
S9-early
S8-late
S8-early
AP
DV
OreR
STYrnai
EGFRrnai
OreR
Sty-RNAi
EGFR-RNAi
